# Interactions between genetic and lifestyle factors on cardiometabolic disease-related outcomes in Latin American and Caribbean populations: A systematic review

**DOI:** 10.3389/fnut.2023.1067033

**Published:** 2023-01-26

**Authors:** Ramatu Wuni, Eduard F. Ventura, Katherine Curi-Quinto, Claudia Murray, Richard Nunes, Julie A. Lovegrove, Mary Penny, Marta Favara, Alan Sanchez, Karani Santhanakrishnan Vimaleswaran

**Affiliations:** ^1^Hugh Sinclair Unit of Human Nutrition, Department of Food and Nutritional Sciences and Institute for Cardiovascular and Metabolic Research (ICMR), University of Reading, Reading, United Kingdom; ^2^Instituto de Investigación Nutricional, Lima, Peru; ^3^Department of Real Estate and Planning, University of Reading, Reading, United Kingdom; ^4^Oxford Department of International Development, University of Oxford, Oxford, United Kingdom; ^5^Grupo de Análisis para el Desarrollo (GRADE), Lima, Peru; ^6^Institute for Food, Nutrition and Health (IFNH), University of Reading, Reading, United Kingdom

**Keywords:** systematic review, nutrigenetics, Latin American and Caribbean, genetics, gene-lifestyle interaction, dietary intake, physical activity

## Abstract

**Introduction:**

The prevalence of cardiometabolic diseases has increased in Latin American and the Caribbean populations (LACP). To identify gene-lifestyle interactions that modify the risk of cardiometabolic diseases in LACP, a systematic search using 11 search engines was conducted up to May 2022.

**Methods:**

Eligible studies were observational and interventional studies in either English, Spanish, or Portuguese. A total of 26,171 publications were screened for title and abstract; of these, 101 potential studies were evaluated for eligibility, and 74 articles were included in this study following full-text screening and risk of bias assessment. The Appraisal tool for Cross-Sectional Studies (AXIS) and the Risk Of Bias In Non-Randomized Studies—of Interventions (ROBINS-I) assessment tool were used to assess the methodological quality and risk of bias of the included studies.

**Results:**

We identified 122 significant interactions between genetic and lifestyle factors on cardiometabolic traits and the vast majority of studies come from Brazil (29), Mexico (15) and Costa Rica (12) with FTO, APOE, and TCF7L2 being the most studied genes. The results of the gene-lifestyle interactions suggest effects which are population-, gender-, and ethnic-specific. Most of the gene-lifestyle interactions were conducted once, necessitating replication to reinforce these results.

**Discussion:**

The findings of this review indicate that 27 out of 33 LACP have not conducted gene-lifestyle interaction studies and only five studies have been undertaken in low-socioeconomic settings. Most of the studies were cross-sectional, indicating a need for longitudinal/prospective studies. Future gene-lifestyle interaction studies will need to replicate primary research of already studied genetic variants to enable comparison, and to explore the interactions between genetic and other lifestyle factors such as those conditioned by socioeconomic factors and the built environment. The protocol has been registered on PROSPERO, number CRD42022308488.

**Systematic review registration:**

https://clinicaltrials.gov, identifier CRD420223 08488.

## 1. Introduction

Cardiometabolic diseases such as hypertension and type 2 diabetes (T2D) are accountable for most non-communicable disease (NCD) deaths and impose an economic burden on low- and middle-income countries (1). In Latin American and the Caribbean populations (LACP), the prevalence of hypertension, T2D and obesity is 47, 22, and above 20%, respectively (2, 3). The etiology of cardiometabolic diseases is multifactorial where studies have demonstrated an interaction between the environment, genetic, behavioral, physiological, and socioeconomic factors (4–9). These intertwined mechanisms interact, modifying the risk of developing cardiometabolic diseases. Genetic variations or single nucleotide polymorphisms (SNPs) may modify the susceptibility to cardiometabolic diseases conditioned by the exposure to lifestyle factors (4, 5). Genome-wide association studies have identified several genetic loci associated with cardiometabolic traits but most of these studies have been performed in Caucasian populations (10–15). Similarly, majority of nutrigenetic studies have been performed in Western countries and the findings might not be applicable to low-income countries due to variations in allele frequencies, dietary pattern, and environmental factors (5, 16).

Factors such as changes in patterns of food consumption, the process of urbanization, increased health and socioeconomic disparities, underfinanced healthcare systems, lower levels of income and productivity, and the rise in sedentary lifestyle have led to an increase in NCDs (17–21). Moreover, studies have shown that metabolic responses to lifestyle factors such as diet and physical activity vary between ethnicities due to genetic heterogeneity (4, 5, 22, 23), and hence we sought to determine which lifestyle factors are interacting with genetic variants in different LACP with regards to cardiometabolic disease traits. The discovery of gene-lifestyle interactions in LACP will help to identify population subgroups that will respond to lifestyle interventions.

The influence of gene-lifestyle interactions on obesity, T2D and cardiovascular diseases (CVDs) has been broadly studied, and there is evidence that the genetic risk of cardiometabolic traits can be modified (4, 5, 24–27). However, to our knowledge, no previous systematic reviews have been conducted regarding the interactions of genetic and lifestyle factors on cardiometabolic disease traits in LACP. Thus, the objective of this systematic review was to identify studies examining the interactions between genetic variants and lifestyle factors such as diet, nutrient intake, nutritional status, physical activity, socioeconomic factors, and the built environment on obesity, CVDs, and T2D-related traits in LACP.

## 2. Methods

### 2.1. Inclusion and exclusion criteria

Eligible for inclusion were articles that explored the interaction between genetic variations and lifestyle factors on cardiometabolic disease traits in LACP. All cardiometabolic diseases and traits were considered including CVDs, cerebrovascular diseases such as stroke, blood lipid levels, obesity-related traits such as body mass index (BMI) and T2D-related traits such as fasting glucose. The eligible articles included observational and dietary intervention studies and were in either English, Spanish, or Portuguese. Articles that did not explore gene-lifestyle interactions or were not based on LACP were excluded.

### 2.2. Information sources and search strategy

A literature search was conducted in MEDLINE (*via* PubMed and EBSCO Host), Web of Science, ScienceDirect, SciELO, SCOPUS, Taylor & Francis Online, Cochrane library, LILACS (Latin American and Caribbean Health Sciences Literature), IBECS, Google Scholar, and ERIC (Education Resources Information Center *via* EBSCO Host) search engines until the 25th of May 2022. To reach literature saturation, the researchers conducted independent search strings (Supplementary Table 1), and the included publications were searched through to identify potential articles in reference lists. We followed the Peer Review of Electronic Search Strategies (PRESS) guideline (28) and the literature search was limited to human participants and had no dates of publication restrictions. The protocol was registered on PROSPERO, number CRD42022308488.

### 2.3. Study selection, synthesis methods, effect measures, and data collection process

**FIGURE 1:**
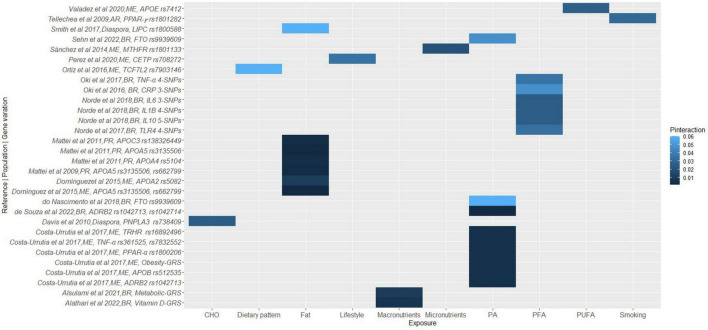
A heat map showing the findings for gene-lifestyle interactions on overall cardiometabolic disease risk. Alsulami et al. (72), Metabolic-GRS = *TCF7L2 (rs12255372, rs7903146); MC4R (rs17782313, rs2229616); PPAR γ (rs1801282); FTO (rs8050136); CDKN2A/2B (rs10811661); KCNQ1 (rs2237892); CAPN10 (rs5030952);* Alathari et al. (73), Vitamin D-GRS = *VDR (rs2228570, rs7975232), DHCR7 (rs12785878), CYP2R1(rs12794714), CYP24A1(rs6013897), GC (rs2282679), FTO (rs8050136, rs10163409), TCF7L2 (rs12255372, rs7903146), MC4R (rs17782313), KCNQ1 (rs2237895, rs2237892), CDKN2A (rs10811661), PPAR γ (rs1801282), CAPN10 (rs5030952);* Costa-Urrutia et al. (118), Obesity-GRS = *ABCA1 (rs2230806, rs9282541); ADIPOQ (rs2241766); ADRB2 (rs1042713); AGT (rs699); APOA4 (rs675); APOB (rs512535); APOE (rs405509); CAPN10 (rs2975760, rs2975762, rs3792267); FTO (rs1121980, rs9939609); HNF4 (rs745975); LIPC (rs1800588); LPL (rs320); PPAR-*α *(rs1800206); PPAR-γ (rs1801282); SCARB1 (rs1084674); TCF7L2 (rs7903146); TNF (rs361525); TRHR (rs1689249, rs783255*2); Norde et al. (79), 5-SNPs = *IL10 rs1554286, rs1800871, rs1800872, rs1800890, rs3024490*; Oki et al. (78), 4-SNPs = *TNF-*α *rs1799724, rs1800629, rs361525, rs1799964;* Norde et al. (76), 4-SNPs = *TLR4 rs11536889, rs4986790, rs4986791, rs5030728;* Oki et al. (77), 3-SNPs = *CRP rs1205, rs1417938, rs2808630;* Norde et al. (79), 4-SNPs = *IL1B rs16944, rs1143623, rs1143627, rs1143643; 3-SNPs* = *rs1800795, rs1800796, rs1800797;* BR, Brazilian; ME, Mexican; PR, Puerto Rican; AR, Argentinian.

**FIGURE 2:**
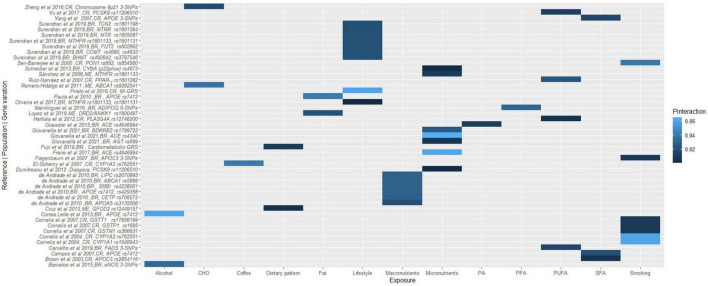
A heat map showing the findings for gene-lifestyle interactions on cardiovascular disease traits. Sotos-Prieto et al. (144), MI-GRS = *CDKN2A/2B (rs4977574, rs10757274, rs2383206, rs1333049); CELSR2-PSRC1-SORT1 (rs646776, rs599839); CXCL12(rs501120, rs1746048); HNF1A, C12orf43 (rs2259816); MRAS (rs9818870); SLC22A3 (rs2048327); LPAL2 (rs3127599); LPA (rs7767084, rs10755578);* Fujii et al. (64), Cardiometabolic-GRS = *APOA5 (rs662799); APOB (rs693, rs1367117); LDLR (rs688, rs5925); LIPC (rs2070895, rs1800588);* Brown et al. (125), 3-SNPs = *APOE rs7412, rs449647, rs429358*; Fiegenbaum et al. (92), 3-SNPs = APOC3 rs2854116, rs2854117, rs5128; Maintinguer Norde et al. (75), 5-SNPs = ADIPOQ rs2241766, rs16861209, rs17300539, rs266729, rs1501299; Carvalho et al. (65), 3-SNPs = FADS rs174575, rs174561, rs3834458; Barcelos et al. (88), 3-SNPs = eNOS rs2070744, rs1799983, rs61722009; Zheng et al. (135), 3-SNPs = Chromosome 9p21 rs4977574, rs2383206, rs1333049. BR, Brazilian; CR, Costa Rican.

**FIGURE 3:**
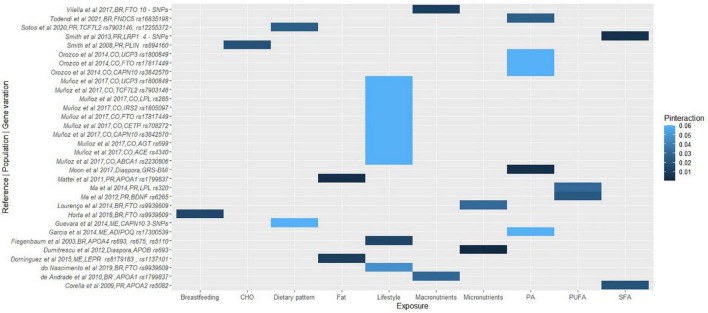
A heat map showing the findings for gene-lifestyle interactions on obesity traits. Vilella et al. (80), 10-SNPs = *FTO rs79149291, rs62048379, rs115530394, rs75066479, rs2003583, rs115662052, rs114019148, rs62034079, rs1123817, rs16952663;* Smith et al. (146), 4-SNPs = *LRP1 rs1799986, rs1799986, rs1800191, rs715948*; Cao et al. (107), 3-SNPs = *CAPN10 rs5030952, rs3792267, rs2975762*. BR, Brazilian; PR, Puerto Rican; ME, Mexican; CO, Colombian.

**FIGURE 4:**
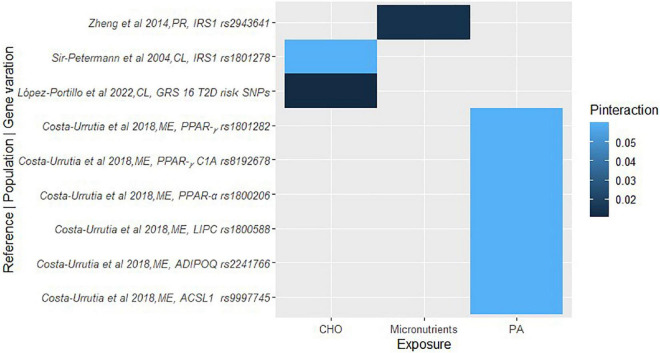
A heat map showing the findings for gene-lifestyle interactions on diabetes traits. López-Portillo et al. (161), GRS-16 Type 2 Diabetes (T2D) risk SNPs = *MTNR1B (rs10830963); TCF7L2 (rs7903146); CDKAL1 (rs7756992); ADCY5 (rs11717195); ANK1 (rs516946); BCAR1 (rs7202877); CDC123 (rs11257655); DUSP9 (rs5945326); GRB14 (rs3923113); RASGRP1 (rs7403531); TLE4 (rs17791513); TLE1 (rs2796441); ZBED3 (rs6878122)*.

Duplicate articles were removed using Rayyan software (29), titles and abstracts were blindly screened to assess against the pre-established inclusion criteria, followed by full-text screening and discussion until consensus between E.F.V. and R.W. All the data required to assess the eligibility of the studies was available, hence study investigators were not contacted to obtain or confirm the data. The reviewers ensured consistency across the data that needed to be extracted, and a narrative synthesis was conducted to collate the data, including populations, lifestyle factors, study designs, genetic variations, cardiometabolic disease traits, and *P*-values for gene-lifestyle interactions on obesity, diabetes and CVD traits. *P*-values for gene-lifestyle interactions were used as indicators of the relationship between the exposure (genetic and lifestyle factors) and the outcome (cardiometabolic traits). *P*-values < 0.05 were considered statistically significant. *P*_interaction_ refers to the *P*-value for the interaction between the genetic variant and dietary/lifestyle factors on cardiometabolic traits. To synthesize the findings, we categorized the outcomes into four categories: obesity, diabetes, CVD, and overall cardiometabolic risk. We then coded the exposures considering major themes; proteins, carbohydrates, fats, and fiber as well as plasma fatty acids, polyunsaturated fatty acids (PUFA), saturated fatty acids (SFA), breastfeeding, smoking, alcohol, coffee, and lifestyle (if the exposure was multiple, including factors embracing diet, physical activity, smoking, and/or socioeconomic status, education), macronutrients (when the exposures included at least proteins, carbohydrates, fats, and fiber), and micronutrients (when the exposure referred to minerals or vitamins). The final graphical representation of the interaction between the genetic variations, and the coded lifestyle factors on the clustered outcomes was a heat map, where the intensity of the color corresponds to the *P*-values of the gene-lifestyle interactions (Figures 1–4). All heat maps were produced using the ggplot2 package (30) in R software with RStudio environment (31). A meta-analysis could not be conducted due to the wide range of dietary factors, genetic variants and cardiometabolic traits investigated by the included studies, in addition to heterogeneity in the methods used.

### 2.4. Data items

Data was extracted in Table 1 and the main outcomes were diabetes, obesity, CVD, and their related traits including lipid levels, blood pressure and anthropometric measurements.

**TABLE 1:** Summary table of gene-lifestyle interactions and study characteristics.

Gene and SNP	Population and sample size	Study design	Dietary/lifestyle factor	Outcome	P_interaction_*	References
**FTO**						
*rs9939609*	Brazilian *n* = 1,088	LS	Plasma vitamin D	BMI	0.02–0.04	Lourenço et al. (84)
*rs9939609*	Brazilian *n* = 1,215	C-S	Screen time	Cardiometabolic risk score	0.047	Sehn et al. (96)
*rs9939609*	Brazilian *n* = 432	C-C	Physical activity intervention	TC, HDL, LDL, TG, glucose, insulin, HOMA-IR, QUICKI	NS	do Nascimento et al. (98)
*rs9939609*	Brazilian *n* = 3,701	P-C	Breastfeeding	BMI, overweight, fat mass, lean mass, WC, visceral, and subcutaneous abdominal fat thickness	0.01–0.02	Horta et al. (102)
*rs9939609*	Brazilian *n* = 434	C-C	hypocaloric diet, physical exercise program	BMI, WC, AC	0.047	do Nascimento et al. (99)
*rs17817449*	Colombian *n* = 212/212	C-C	Physical activity	BMI	NS	Orozco et al. (163)
*rs17817449*	Colombian *n* = 1,081	C-S	Socioeconomic stratum, maternal education year, maternal breastfeeding	BMI	NS	Muñoz et al. (164)
*rs79149291, rs62048379, rs115530394, rs75066479, rs2003583, rs115662052, rs114019148, rs62034079, rs1123817, rs16952663*	Brazilian *n* = 1,191	C-S	Carbohydrate, protein, total fat, MUFA, PUFA:SFA intake	Overweight/obesity	0.01	Vilella et al. (80)
***APOE***						
*rs7412, rs429358*	Brazilian *n* = 567	C-S	Olive oil, PUFA, sucrose, soluble and insoluble fiber	LDL, TG, TC	0.018–0.04	de Andrade et al. (62)
*rs7412*	Brazilian *n* = 252	C-S	Total fat, PUFA: SFA	LDL, TG, VLDL	<0.05	Paula et al. (63)
*rs7412*	Brazilian *n* = 851	P-C	Alcohol intake	SBP, DBP	NS	Correa Leite et al. (89)
*rs7412*	Mexican *n* = 224	C-S	MUFA intake, n-3:n-6	TC, Non-HDL, LDL, HbA1c	0.016–0.035	Torres-Valadez et al. (103)
*rs7412*	Costa Rican *n* = 420	C-S	SFA	TG, TC, VLDL, LDL, HDL, Apo A1, Apo B, LDL particle size	0.02–0.03	Campos et al. (124)
*rs7412, rs429358*, *rs449647*	Costa Rican *n* = 1,927/1,927	C-C	SFA	TC, HDL, LDL, TG, MI	0.0157	Yang et al. (125)
**APOA5**						
*rs3135506*	Brazilian *n* = 567	C-S	Olive oil, PUFA, sucrose, soluble and insoluble fiber	LDL, TG, TC	0.018–0.04	de Andrade et al. (62)
*rs3135506, rs662799*	Mexican *n* = 100/100	C-C	SFA, total fat	TC, TG, LDL, HDL, obesity	0.001–0.02	Domínguez-Reyes et al. (105)
*rs3135506, rs662799*	Puerto Rican *n* = 802	LS	Total fat	WC, serum glucose, SBP, DBP, HDL, LDL, TC, VLDL	0.002–0.032	Mattei et al. (152)
**APOA5**						
*rs3135506*	Puerto Rican *n* = 821	LS	Total fat	WC, SBP, DBP	0.001–0.005	Mattei et al. (147)
**MTHFR**						
*rs1801133, rs1801131*	Brazilian *n* = 3,803	C-S	Physical activity, alcohol intake, and blood folate	Homocysteine	<0.001–0.002	Oliveira et al. (85)
*rs1801133, rs1801131*	Brazilian *n* = 113	C-S	Fat, protein, carbohydrate intake, physical activity	Vitamin B12, homocysteine, folic acid, HDL, LDL, TG, oxidized LDL	0.005–0.034	Surendran et al. (66)
*rs1801133*	Mexican *n* = 996 (women); 231 (new-borns)	P-C	Folate and Vitamin B12	Weight, length and BMI of new-born	0.02	Torres-Sánchez et al. (115)
*rs1801133*	Mexican *n* = 130	C-S	Vitamin B12, alcohol intake	Plasma Folate, total homocysteine	0.01	Torres-Sánchez et al. (116)
**ACE**						
*rs4340*	Brazilian *n* = 335	C-S	Sodium, potassium, calcium, magnesium	SBP, DBP	0.004–0.009	Giovanella et al. (81)
*rs4340*	Colombian *n* = 1,081	C-S	Socioeconomic stratum, maternal education year, maternal breastfeeding	BMI	NS	Muñoz et al. (164)
*rs4646994*	Brazilian *n* = 234	C-C	Sodium	Hypertension	NS	Freire et al. (82)
**ACE**						
*rs4646994*	Brazilian *n* = 34	RCT	Physical activity	SBP, DBP	0.02 –0.002	Goessler et al. (97)
**TCF7L2**						
*rs7903146*	Mexican *n* = 137	P-LS	Two diets: Nopal tortilla and whole grain bread	Weight, BMI, WC, HC, WHR, glucose, HbA1c, TG, TC, HDL, LDL, insulin, HOMA-B, HOMA-IR, GLP-1	NS	López-Ortiz et al. (185)
*rs7903146*	Colombian *n* = 1,081	C-S	Socioeconomic stratum, maternal education year, maternal breastfeeding	BMI	NS	Muñoz et al. (164)
*rs7903146, rs12255372*, *rs7903146, rs12255372*	Puerto Rican *n* = 1,120	C-S	Mediterranean diet score	BMI, WC, weight	0.014–0.036	Sotos-Prieto et al. (150)
**ABCA1**						
*rs5888*	Brazilian *n* = 567	C-S	Olive oil, PUFA, sucrose, soluble and insoluble fiber	LDL, TG, TC	0.018–0.04	de Andrade et al. (62)
*rs9282541*	Mexican *n* = 3,591	C-S	Carbohydrate	HDL	0.037	Romero-Hidalgo et al. (112)
*rs2230806*	Colombian *n* = 1,081	C-S	Socioeconomic stratum, maternal education year, maternal breastfeeding	BMI	NS	Muñoz et al. (164)
**LIPC**						
*rs2070895*	Brazilian *n* = 567	C-S	Olive oil, PUFA, sucrose, soluble and insoluble fiber	LDL, TG, TC	0.018–0.04	de Andrade et al. (62)
*rs1800588*	Mexican *n* = 167/398	C-C	Maximal oxygen consumption (VO2 max), muscle endurance (ME)	pre-diabetes (fasting glucose concentrations)	NS	Costa-Urrutia et al. (119)
*rs1800588*	Dominican/Puerto Rican, other Caribbean Hispanics *n* = 41	RCT	High fat diet	HDL, LDL, TC, TG, glucose	NS	Smith et al. (153)
**APOC3**						
*rs2854116, rs2854117, rs5128*	Brazilian *n* = 673	C-S	Smoking	TG	0.009	Fiegenbaum et al. (92)
*rs2854116, T-625del*	Costa Rican *n* = 336	C-S	SFA	TG, TC, LDL, HDL, Apo B, LDL diameter	0.0004 –0.01	Brown et al. (126)
*rs138326449*	Puerto Rican *n* = 821	LS	Total fat	WC, SBP, DBP	0.001–0.005	Mattei et al. (147)
**CETP**						
*rs708272*	Brazilian *n* = 567	C-S	Olive oil, PUFA, sucrose, soluble and insoluble fiber	LDL, TG, TC	0.018–0.04	de Andrade et al. (62)
*rs708272*	Mexican *n* = 215	C-S	Sucrose intake, physical activity	TC, LDL, TG, HDL, TG:HDL, BMI, WC	0.033–0.037	Campos-Perez et al. (113)
**CETP**						
*rs708272*	Colombian *n* = 1,081	C-S	Socioeconomic stratum, maternal education year, maternal breastfeeding	BMI	NS	Muñoz et al. (164)
**ADIPOQ**						
*rs2241766, rs16861209, rs17300539, rs266729, rs1501299*	Brazilian *n* = 262	C-S	Plasma fatty acids (14:0, 16:0, 16:1 n-7, 18:0, 18:1, 18:2 n-6, 18:3 n-3, 20:3 n-6, AA, EPA, DPA, DHA, SFA, MUFA, n-6, n-3, PUFA, n-3 HUFA, SCD-16, SCD-18, D5D, D6D)	Systemic Inflammation	0.019–0.044	Maintinguer Norde et al. (75)
*rs17300539*	Mexican *n* = 394	C-S	MUFA, physical activity	adiponectin level	NS	Garcia-Garcia et al. (120)
*rs2241766*	Mexican *n* = 167/398	C-C	VO2 max, ME	pre-diabetes (fasting glucose concentrations)	NS	Costa-Urrutia et al. (119)
**PPAR-γ**						
*rs1801282*	Mexican *n* = 167/398	C-C	VO2 max, ME	pre-diabetes (fasting glucose concentrations)	NS	Costa-Urrutia et al. (119)
*rs1801282*	Costa Rican *n* = 1,805/1,805	C-C	PUFA intake	MI, PUFA in adipose tissue	0.016 –0.03	Ruiz-Narváez et al. (127)
**PPAR-γ**						
*rs1801282*	Argentina *n* = 572	C-S	Smoking status	MetS, fasting plasma glucose, SBP, DBP, WC, HDL, TG, fasting insulin, loginsulin, HOMA-IR, LogHOMA-IR, QUICKI	0.031	Tellechea et al. (165)
**PPAR-γ C1A**						
rs8192678	Mexican *n* = 167/398	C-C	VO2 max, ME	pre-diabetes (fasting glucose concentrations)	NS	Costa-Urrutia et al. (119)
**PPAR-α**						
rs1800206	Mexican *n* = 167/398	C-C	VO2 max, ME	pre-diabetes (fasting glucose concentrations)	NS	Costa-Urrutia et al. (119)
rs1800206	Mexican *n* = 608	C-C	VO2 max, ME	BMI, WC, fat mass, pre-DM	0.001–0.007	Costa-Urrutia et al. (118)
**APOA4**						
*rs693, rs675, rs5110*	Brazilian *n* = 391	C-S	Smoking, alcohol intake, physical activity	BMI, WC	0.007–0.02	Fiegenbaum et al. (91)
*rs5104*	Puerto Rican *n* = 821	LS	Total fat	WC, SBP, DBP	0.001–0.005	Mattei et al. (147)
**IRS1**						
*rs2943641*	Puerto Rican *n* = 1,144	LS	25(OH)D	HOMA-IR	0.004–0.023	Zheng et al. (159)
*rs1801278*	Chile *n* = 243	NRCT	3-day unrestricted diet containing 300 g/d of carbohydrate, an overnight fast of 10 h and 75 g glucose	Fasting glucose, fasting insulin, fasting HOMA-IR, insulinogenic index, insulin sensitivity index composite	NS	Sir-Petermann et al. (162)
**IRS2**						
*rs1805097*	Colombian *n* = 1,081	C-S	Socioeconomic stratum, maternal education year, maternal breastfeeding	BMI	NS	Muñoz et al. (164)
**PON1**						
*rs662*	Mexican *n* = 206	C-S	Urinary 1-hydroxypyrene	Serum asymmetric dimethylarginine (ADMA)	0.02	Ochoa-Martínez et al. (121)
*rs662*	Mexican *n* = 185	C-S	Urinary arsenic levels	ADMA, fatty acid-binding protein 4, micro-RNAs	< 0.001 – < 0.010	Ochoa-Martínez et al. (122)
*rs662, rs854560*	Costa Rican *n* = 492/518	C-C	Smoking status	MI	0.04	Sen-Banerjee et al. (138)
**AGT**						
*rs699*	Brazilian *n* = 335	C-S	Sodium, potassium, calcium, magnesium	SBP, DBP	0.004–0.009	Giovanella et al. (81)
*rs699*	Colombian *n* = 1,081	C-S	Socioeconomic stratum, maternal education year, maternal breastfeeding	BMI	NS	Muñoz et al. (164)
**ADRB2**						
*rs1042713, rs1042714*	Brazilian *n* = 197	P-C	Physical exercise intervention	Body fat, AC, BMI, DBP, SBP, TC, HDL, LDL, TG, glucose, insulin, HOMA-IR, QUICK, TG-glucose index	0.001	de Souza et al. (94)
rs1042713	Mexican *n* = 608	C-C	VO2 max, ME	BMI, WC, fat mass, pre-DM	0.001–0.007	Costa-Urrutia et al. (118)
**TNF-α**						
*rs1799724, rs1800629, rs361525, rs1799964*	Brazilian *n* = 281	C-S	Plasma fatty acids (C14:0, C16:0, C18:0, C16:1, C18:1, n-6, C18:2, C20:3, C20:4, n-3, C18:3, C20:5, C22:5, C22:6, n-3 HUFA, SCD-16, SCD-18, D5D, D6D, n-6:n-3, SFA, MUFA, PUFA)	Systemic inflammation	0.026 –0.044	Oki et al. (78)
**TNF-α**						
rs361525, rs7832552	Mexican *n* = 608	C-C	VO2 max, ME	BMI, WC, fat mass, pre-diabetes	0.001–0.007	Costa-Urrutia et al. (118)
**CAPN10**						
rs5030952, rs3792267, rs2975762	Mexican *n* = 31	P-C	Low SFA diet, soy protein, soluble fiber	TC, TG, HDL, LDL	NS	Guevara-Cruz et al. (107)
*rs3842570*	Colombian *n* = 212/212	C-C	Physical activity	BMI	NS	Orozco et al. (163)
*rs3842570*	Colombian *n* = 1,081	C-S	Socioeconomic stratum, maternal education year, maternal breastfeeding	BMI	NS	Muñoz et al. (164)
**PCSK9**						
*rs11206510*	Costa Rican *n* = 1,932/2,055	C-C	LC n-3 PUFA, EPA, DPA, DHA	MI	0.012	Yu et al. (128)
*rs11206510*	Mexican American *n* = 1,734	C-S	Serum Vitamin A	LDL	7.65 × 10^–5^	Dumitrescu et al. (158)
**CYP1A2**						
*rs762551*	Costa Rican *n* = 2,014/2,014	C-C	Coffee intake	MI	0.04	El-Sohemy et al. (136)
*rs762551*	Costa Rican *n* = 873/932	C-C	Smoking	MI	NS	Cornelis et al. (139)
**CYP1A1**						
*rs1048943*	Costa Rican *n* = 873/932	C-C	Smoking	MI	NS	Cornelis et al. (139)
**APOA2**						
rs5082	Mexican *n* = 100/100	C-C	SFA, total fat	TC, TG, LDL, HDL, obesity	0.001–0.02	Domínguez-Reyes et al. (105)
rs5082	Puerto Rican *n* = 930	C-S	SFA	BMI	0.02	Corella et al. (145)
**APOA1**						
*rs1799837*	Puerto Rican *n* = 821	LS	Total fat	WC, SBP, DBP	0.001–0.005	Mattei et al. (147)
*rs1799837*	Brazilian *n* = 567	C-S	Olive oil, PUFA, sucrose, soluble and insoluble fiber	LDL, TG, TC	0.018–0.04	de Andrade et al. (62)
**APOB**						
rs512535	Mexican *n* = 608	C-C	VO2 max, ME	BMI, WC, fat mass, pre-DM	0.001–0.007	Costa-Urrutia et al. (118)
*rs693*	Mexican American *n* = 1,734	C-S	Serum Vitamin E	LDL	8.94 × 10^–7^	Dumitrescu et al. (158)
**LPL**						
*rs320*	Puerto Rican *n* = 1,171	LS	Low PUFA, n-3 PUFA, n-6 PUFA intake	BMI, WC	0.02–0.04	Ma et al. (148)
*rs285*	Colombian *n* = 1,081	C-S	Socioeconomic stratum, maternal education year, maternal breastfeeding	BMI	NS	Muñoz et al. (164)
**UCP3**						
*rs1800849*	Colombian *n* = 212/212	C-C	Physical activity	BMI	NS	Orozco et al. (163)
*rs1800849*	Colombian *n* = 1,081	C-S	Socioeconomic stratum, maternal education year, maternal breastfeeding	BMI	NS	Muñoz et al. (164)
***TLR4*** *rs11536889, rs4986790*, *rs4986791, rs5030728*	Brazilian *n* = 262	C-S	Systemic Inflammation	0.034	Norde et al. (76)	Systemic inflammation
***BDKRB2*** *rs1799722*	Brazilian *n* = 335	C-S	Sodium, potassium, calcium, magnesium	SBP, DBP	0.004–0.009	Giovanella et al. (81)
***FADS*** *rs174575, rs174561, rs3834458*	Brazilian *n* = 250	C-S	α-linolenic acid, linoleic:α-linolenic acid ratio.	Plasma concentration of PUFA	0.004–0.028	Carvalho et al. (65)
***CYBA (p22phox)*** *rs4673*	Brazilian *n* = 1,298	C-S	Urinary sodium	SBP, DBP, hypertension	<0.001–0.004	Schreiber et al. (83)
***eNOS*** *rs2070744, rs1799983, rs61722009*	Brazilian *n* = 113	C-S	Alcohol intake	SBP, DBP, nitrite levels in plasma	0.033	Barcelos et al. (88)
***FNDC5*** *rs16835198*	Brazilian *n* = 1,701	C-S	Cardiorespiratory fitness, lower limb strength	WC, BMI	0.007–0.044	Todendi et al. (95)
***LEPR*** rs8179183, rs1137101	Mexican *n* = 100/100	C-C	SFA, total fat intake	TC, TG, LDL, HDL, obesity	0.001–0.02	Domínguez-Reyes et al. (105)
***ACSL1*** rs9997745	Mexican *n* = 167/398	C-C	VO2 max, ME	pre-diabetes	NS	Costa-Urrutia et al. (119)
***TRHR*** rs16892496	Mexican *n* = 608	C-C	VO2 max, ME	BMI, WC, fat mass, pre-diabetes	0.001–0.007	Costa-Urrutia et al. (118)
***DRD2/ANKK1*** rs1800497	Mexican *n* = 175	C-S	Maltose, total fat, MUFA, dietary cholesterol	**TG**	0.001 –0.041	Ramos-Lopez et al. (104)
***GFOD2*** rs12449157	Mexican *n* = 41	P-C	Low SFA diet, soy protein and soluble fiber	TC, LDL, HDL, TG	0.002–0.006	Guevara-Cruz et al. (106)
***PLA2G4A*** *rs12746200*	Costa Rican *n* = 1,936/2,035	C-C	n-6 PUFA intake	MI	0.005	Hartiala et al. (129)
***CRP*** *rs1205, rs1417938, rs2808630*	Brazilian *n* = 262	C-S	Plasma fatty acids (Myristic acid, Palmitic acid, Stearic acid, C16:1, C18:1, n-6, C18:2, C20:3, C20:4, n-3, C18:3, C20:5, C22:5, C22:6, n-3 HUFA, SFA, MUFA, PUFA, SCD-16, SCD-18, D5D, D6D, n-6/n-3)	Systemic Inflammation	0.047	Oki et al. (77)
***GSTM1*** *rs366631* ***GSTP1*** *rs1695* ***GSTT1*** *rs17856199*	Costa Rican *n* = 2,042/2,042	C-C	Cruciferous vegetables, smoking	MI	0.008	Cornelis et al. (137)
***IL1B*** *rs16944, rs1143623, rs1143627, rs1143643* ***IL6*** *rs1800795, rs1800796, rs1800797* ***IL10*** *rs1554286, rs1800871, rs1800872, rs1800890, rs3024490*	Brazilian *n* = 301	C-S	Plasma fatty acid (C14:0, C16:0, C16:1 n-9, C18:0, C18:1 n-9, C18:2 n-6, C18:3 n-3, AA, EPA, DHA, n-6, n-3); desaturates activity (SCD-16, SCD-18, D6D, D5D)	MetS	0.007–0.043	Norde et al. (79)
***MTR*** *rs1805087* ***MTRR*** *rs1801394* ***TCN2*** *rs1801198* ***COMT*** *rs4680, rs4633* ***BHMT*** *rs492842, rs3797546* ***FUT2*** *rs602662*	Brazilian *n* = 113	C-S	Fat, protein, carbohydrate intake, physical activity	Vitamin B12, homocysteine, folic acid, HDL, LDL, triglycerides, oxidized LDL	0.005–0.034	Surendran et al. (66)
***GSTM1*** *rs366631* ***GSTP1*** *rs1695* ***GSTT1*** *rs17856199*	Costa Rican *n* = 2,042/2,042	C-C	Cruciferous vegetables, smoking	MI	0.008	Cornelis et al. (137)
***LRP1*** *rs1799986, rs1799986*, *rs1800191, rs715948*	Puerto Rican *n* = 676	P-C	SFA, palmitic acid (C16:0), stearic acid (C18:0), butyric acid (C4:0), caproic acid (C6:0), caprylic acid (C8:0), capric acid (C10:0), lauric acid (C12:0), myristic acid (C14:0)	BMI, WC, HC	0.002–0.004	Smith et al. (146)
***PLIN*** *rs894160*	Puerto Rican *n* = 920	LS	Complex carbohydrate, total carbohydrate, simple sugars	WC, HC, BMI	0.004–0.035	Smith et al. (155)
***Chromosome 9p21*** *rs4977574, rs4977574*, *rs2383206, rs1333049*	Costa Rican *n* = 1,560/1,751	C-C	Sugar sweetened beverages, fruit juice	MI	0.005–0.03	Zheng et al. (135)
***BDNF*** *rs6265*	Puerto Rican *n* = 1,340	LS	PUFA, n-3: n-6, food intake	BMI, WC, HC	0.002–0.043	Ma et al. (149)
***PNPLA3*** *rs738409*	Hispanic ancestry *n* = 153	C-S	Carbohydrate, sugar	Hepatic fat	0.01–0.04	Davis et al. (156)
***SRBI*** *rs4238001*	Brazilian *n* = 567	C-S	Olive oil, PUFA, sucrose, soluble and insoluble fiber	LDL, TG, TC	0.018–0.04	de Andrade et al. (62)
***GRS*** *:TCF7L2 (rs12255372, rs7903146); MC4R (rs17782313, rs2229616); PPARγ (rs1801282); FTO (rs8050136); CDKN2A/2B (rs10811661); KCNQ1 (rs2237892); CAPN10 (rs5030952)*	Brazilian *n* = 200	C-S	Total fat, SFA, PUFA, MUFA, carbohydrate, protein	HbA1c, HOMA-IR, HOMA-B, fasting glucose, fasting insulin, insulin:glucose, body fat mass, BMI, WC	0.002–0.017	Alsulami et al. (72)
**GRS:** *VDR (rs2228570, rs7975232), DHCR7 (rs12785878), CYP2R1(rs12794714), CYP24A1(rs6013897), GC (rs2282679), FTO (rs8050136, rs10163409), TCF7L2 (rs12255372, rs7903146), MC4R (rs17782313), KCNQ1 (rs2237895, rs2237892), CDKN2A (rs10811661), PPARγ (rs1801282), CAPN10 (rs5030952)*	Brazilian *n* = 187	C-S	Carbohydrate, protein, fat and fiber	BMI, WC, body fat, glucose, HbA1c, fasting insulin	0.006	Alathari et al. (73)
***GRS****: ABCA1 (rs2230806, rs9282541); ADIPOQ (rs2241766); ADRB2 (rs1042713); AGT (rs699); APOA4 (rs675); APOB (rs512535); APOE (rs405509); CAPN10 (rs2975760, rs2975762, rs3792267); FTO (rs1121980, rs9939609); HNF4 (rs745975); LIPC (rs1800588); LPL (rs320); PPAR-*α *(rs1800206); PPAR-γ (rs1801282); SCARB1 (rs1084674); TCF7L2 (rs7903146); TNF (rs361525); TRHR (rs1689249, rs7832552)*	Mexican *n* = 608	C-C	VO2 max, ME	BMI, WC, fat mass, pre-diabetes	0.001–0.007	Costa-Urrutia et al. (118)
***GRS:*** *CDKN2A/2B (rs4977574, rs10757274, rs2383206, rs1333049); CELSR2-PSRC1-SORT1 (rs646776, rs599839); CXCL12(rs501120, rs1746048); HNF1A, C12orf43 (rs2259816); MRAS (rs9818870); SLC22A3 (rs2048327); LPAL2 (rs3127599); LPA (rs7767084, rs10755578)*	Costa Rican *n* = 1,534/1,534	C-C	Lifestyle cardiovascular risk score (unhealthy diet, physical inactivity, smoking, elevated waist:hip ratio, high alcohol intake, low socioeconomic status.)	MI	NS	Sotos-Prieto et al. (144)
GRS based on 97 BMI associated SNPs	Puerto Rican, Mexicans, Dominicans, Cuban, Central American, South American *n* = 9,645	P-C	Total physical activity, physical activity at a moderate to vigorous intensity, sedentary behavior	BMI, fat mass, fat mass index, fat percentage, WC Fat-free mass	0.001 –0.005	Moon et al. (160)
GRS: *MTNR1B* (*rs10830963*)*; TCF7L2* (*rs7903146*)*; CDKAL1* (*rs7756992*); *ADCY5* (*rs11717195*); *ANK1* (*rs516946*)*; BCAR1* (*rs7202877*); *CDC123* (*rs11257655*)*; DUSP9 (rs5945326*)*; GRB14* (*rs3923113*)*; RASGRP1* (*rs7403531*)*; TLE4* (*rs17791513*); *TLE1* (*rs2796441*); *ZBED3* (*rs6878122*)	Chile *n* = 2,828	P-C	Sugar sweetened beverages intake	Fasting glucose	0.001–0.02	López-Portillo et al. (161)
***GRS***: *APOA5 (rs662799); APOB (rs693, rs1367117); LDLR (rs688, rs5925); LIPC (rs2070895, rs1800588)*	Brazilian *n* = 228	C-S	Brazilian Healthy Eating Index Revised	Dyslipidaemia	0.001 –0.019	Fujii et al. (64)

*APOE*, Apolipoprotein E; *APOA*, Apolipoprotein A; *ApoB*, Apolipoprotein B; *SRBI*, scavenger receptor class B member 1; *ABCA1*, ATP binding cassette subfamily A member 1; *CETP*, cholesteryl ester transfer protein; *APOC3*, Apolipoprotein C; *ADIPOQ*, adiponectin; *TLR4*, toll like receptor 4; *FTO*, alpha-ketoglutarate dependent dioxygenase; *CRP*, C-reactive protein; *GRS*, genetic risk score; *MTHFR*, methylenetetrahydrofolate reductase; *FADS*, fatty acid desaturase; *TNF*, tumor necrosis factor; *ADRB*, adrenoceptor beta; *ACE*, angiotensin I converting enzyme; *AGT*, angiotensinogen; *BDKRB*, bradykinin receptor; *eNOS*, endothelial nitric oxide synthase; *CYBA*, cytochrome B-245 alpha chain; *IL*, interleukin; *FNDC5*, fibronectin type III domain containing 5; *GRS*, genetic risk score; *VDR*, vitamin D receptor; *DHCR7*, 7-dehydrocholesterol reductase; *CYP2R1*, cytochrome P450 family 2 subfamily R member 1; *CYP24A1*, cytochrome P450 family 24 subfamily A member 1; *GC*, group-specific component; *TCF7L2*, transcription factor 7 like 2; *MC4R*, melanocortin-4-receptor; *KCNQ1*, potassium voltage-gated channel subfamily Q member 1; *CDKN*, cyclin dependent kinase inhibitor; *PPAR*, peroxisome proliferator activated receptor; *CAPN*, Calpain; *MTR*, methionine synthase; *MTRR*, 5-methyltetrahydrofolate-homocysteine methyltransferase reductase; *TCN2*, transcobalamin 2; *COMT*, catechol-O-methyltransferase; *BHMT*, betaine-homocysteine S-methyltransferase; *FUT2* fucosyltransferase 2; *LEPR*, leptin receptor; *TRHR*, thyrotropin releasing hormone receptor; *LIPC*, hepatic lipase; *ACSL*, acyl-CoA synthetase long chain family member 1; *GFOD2*, Glucose-Fructose Oxidoreductase Domain Containing 2; *PCSK9*, proprotein convertase subtilisin/kexin type 9; *PON1* Paraoxonase 1; *CYP1A2*, cytochrome P450 family 1 subfamily A member 2; *PLA2G4A*, phospholipase A2 group IVA; *GSTM1*, glutathione S-transferase Mu 1; *GSTP1*, glutathione S-transferase Pi 1; *GSTT1*, glutathione S-transferase theta 1; *CYP1A1*, cytochrome P450 family 1 subfamily A member 1; *CELSR2*, Cadherin EGF LAG seven-pass G-type receptor 2; *PSRC1*, proline and serine rich coiled-coil 1; *SORT1*, sortilin 1; *CXCL12*, C-X-C motif chemokine ligand 12; *HNF1A*, hepatocyte nuclear factor 1; *MRAS*, muscle RAS oncogene homolog; *SLC22A3*, solute carrier family 22 member 3; *LPAL2*, lipoprotein(A) like 2, pseudogene; *LPA*, lipoprotein(A); *IRS*, insulin receptor substrate; *MTNR1B*, melatonin receptor 1B; *CDKAL1*, CDK5 regulatory subunit-associated protein 1-like 1; *ADCY5*, adenylyl cyclase type V; *ANK1*, ankyrin-1; *BCAR1*, breast cancer anti-estrogen resistance protein 1; *CDC123*, cell division cycle 123; *DUSP9*, dual specificity phosphatase 9; *GRB14*, growth factor receptor bound protein 14; *RASGRP1*, RAS guanyl-releasing protein 1; *TLE*, transducin-like enhancer protein; *ZBED3*, zinc finger BED-Type containing 3; *UCP3*, uncoupling protein 3; *LPL*, lipoprotein lipase; *MetS*, metabolic syndrome; *SBP*, systolic blood pressure; *DBP*, diastolic blood pressure; *WC*, waist circumference; *BMI*, body mass index; *TG*, triglycerides. *HDL*, high-density lipoprotein cholesterol; *HOMA-IR*, homeostasis model assessment estimate of insulin resistance; *QUICKI*, quantitative insulin-sensitivity check index; *AUC*, area under the curve; *TC*, total cholesterol; *VLDL*, very-low density lipoprotein cholesterol; *LDL*, low-density lipoprotein cholesterol. *MI*, myocardial infarction; *PUFA*, polyunsaturated fatty acid. *MUFA*, monounsaturated fatty acid; *SFA*, saturated fatty acid; n-3, omega-3; *LC*, long-chain; *EPA*, eicosapentaenoic acid; *DPA*, docosapentaenoic acid; *DHA*, docosahexaenoic acid; *C-S, C-S*, cross-sectional; *RCT*, randomized controlled trial; *NRCT*, non-randomized controlled trial; *P-C*, prospective cohort; *LS*, longitudinal study; *C-C*, case-control; *NS*, not significant. *Only significant *P_interaction_* values are given.

### 2.5. Risk of bias and certainty of assessment

To evaluate the methodological quality and risk of bias (RoB) of cross-sectional studies we used the Appraisal tool for Cross-Sectional Studies (AXIS) (32) (Supplementary Tables 2, 3). Cohort studies, case-control studies, and non-randomized trials were assessed by using the RoB in Non-randomized Studies—of Interventions (ROBINS-I) assessment tool (32, 33) (Supplementary Table 4). Risk of bias due to missing results was assessed using the AXIS RoB (questions 12–14) and the ROBINS-I assessment [part 5 (questions 5.1–5.4)]. The current article adheres to the recommendations of the Synthesis without Meta-analysis (SWiM) in Systematic Reviews: Reporting Guideline (34).

## 3. Results

### 3.1. Study selection and characteristics

**FIGURE 5:**
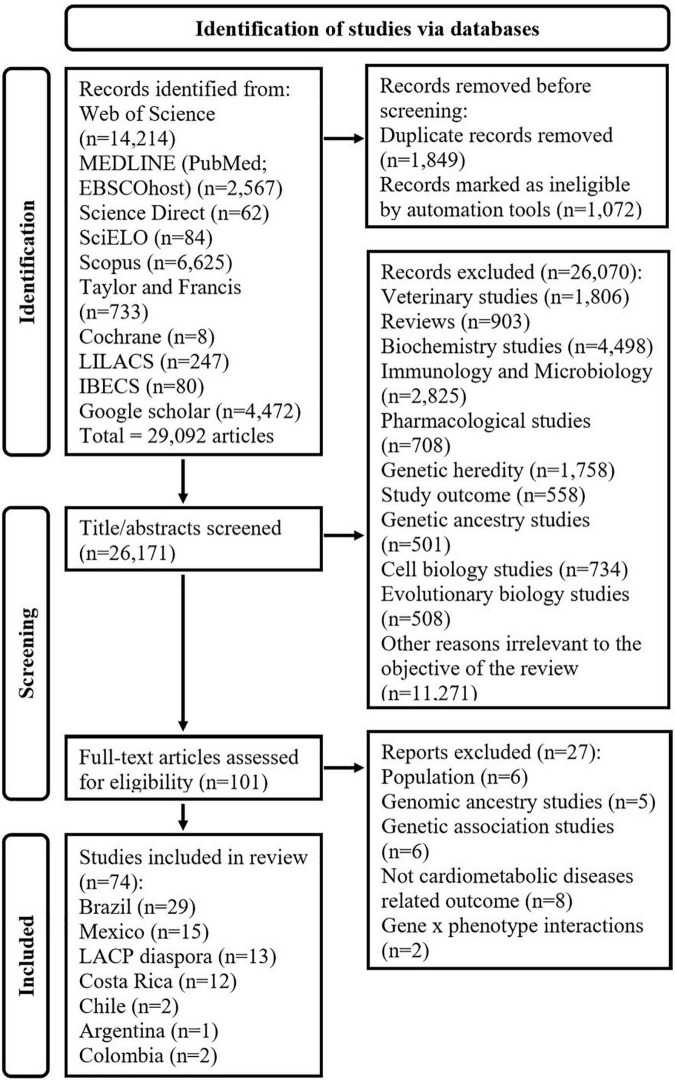
Flow chart showing the exclusion criteria and selection of studies. Literature search was conducted in MEDLINE (*via* PubMed and EBSCO Host), Web of Science, ScienceDirect, SciELO, SCOPUS, Taylor & Francis Online, Cochrane library, LILACS (Latin American and Caribbean Health Sciences Literature), IBECS, Google Scholar, and ERIC (Education Resources Information Center *via* EBSCO Host) search engines until the 25th of May 2022.

The search string results had an output of 29,092 articles and from these, 101 articles were identified as potential studies. After the full-text screening, 27 articles were excluded for the following reasons: six studies were not based on LACP (35–40), five studies aimed to identify the effect of genomic ancestry (41–45), six studies focused only on genetic associations (46–51), eight studies did not include cardiometabolic diseases (52–59), and two studies investigated gene x phenotype interactions (60, 61) as shown in Figure 5. Finally, after excluding the irrelevant articles based on the exclusion criteria, 74 studies were included in this systematic review as shown in Table 1.

### 3.2. Gene-lifestyle interactions in LACP

The 74 studies conducted in LACP encompass ethnicities from Argentina, Colombia, Chile, Costa Rica, Mexico, Brazil, and LACP diaspora, including Dominicans, Puerto Ricans, Mexicans, and other Hispanic ethnicities residing in the United States of America (USA). Most of the studies are focused on four countries: Brazil (29), Mexico (15), Costa Rica (12), and Puerto Ricans in Boston (10). The studies have identified 122 significant gene-lifestyle interactions on cardiometabolic traits (*p* < 0.05), as shown in Table 1. The results are stratified by country to enable identification of ethnic-specific gene-lifestyle interactions and to present a structured mapping of the research gaps for a multidisciplinary audience.

### 3.3. Gene x lifestyle interactions in Brazilians

#### 3.3.1. Interaction between dietary fat intake and genetic variants on CVD traits

Interaction between dietary fat intake and genetic variants on CVD-related traits was examined by five Brazilian studies (62–66). In a cross-sectional study of 567 participants (62), a significant interaction was reported between olive oil intake and Apolipoprotein E (*APOE*) genotype on low-density lipoprotein cholesterol (LDL) (*P*_interaction_ = 0.028), where a high intake of olive oil (≥ once a week) was associated with lower LDL levels in men carrying the “ε*2*” allele but had no effect in men without the “ε*2*” allele. In this study (62), a high polyunsaturated fatty acid (PUFA) intake (> twice a week) was associated with increased LDL levels in carriers of the “ε*4*” allele, but this was not observed in participants without the “ε*4*” allele (*P*_interaction_ = 0.04). A reduction in triglyceride levels in response to a high PUFA intake was also observed in carriers of the “ε*2*” allele but not in participants without the “ε*2*” allele (*P*_interaction_ = 0.04). A high PUFA intake was also associated with increased high-density lipoprotein cholesterol (HDL) concentration in participants without the “ε*4*” allele and reduced HDL levels in carriers of the “ε*4*” allele (*P*_interaction_ = 0.018) (62). In contrast, a cross-sectional study of 252 Brazilian women (63) observed increased triglyceride and very-low density lipoprotein cholesterol (VLDL) in response to a low PUFA or a high fat diet intake in carriers of the “ε*4*” allele of *APOE*, but not in non-carriers (*P*_interaction_ < 0.05 for both). The findings of the first study (62) indicate that, PUFA intake might be beneficial in increasing HDL levels in individuals without the “ε*4*” allele, while in those with the “ε*4*” allele, PUFA intake might contribute to a rise in triglyceride and LDL levels which is associated with higher risk of CVDs (67). Nonetheless, the findings of the second study (63) suggest a detrimental effect of low PUFA intake in carriers of the “ε*4*” allele. The differences in the findings could be attributed to the small sample sizes and the fact that, the second study (63) was conducted in women unlike the first study (62). PUFA is a ligand for peroxisome proliferator-activated receptors (PPARs) which are involved in regulating several lipid-pathway genes and it has been suggested that, increased consumption of PUFA might promote the expression of APOE and hepatic uptake of “ε*4*”-containing VLDL particles (68, 69).

Furthermore, a cross-sectional study of 228 Brazilian participants from the Health Survey of São Paulo (HS-SP) (64) observed significant interactions between a GRS based on seven SNPs (Table 1) and the Brazilian Healthy Eating Index Revised (BHEI-R) on the risk of dyslipidaemia. Participants with a higher GRS (5–8) had a lower odds ratio for dyslipidaemia with an intake of BHEI-R oil component above the median (*P*_interaction_ = 0.019); while those with a GRS > 9 had a lower odds ratio for dyslipidaemia with an intake of BHEI-R solid fats, alcoholic beverages and added sugars (SoFAAS) component below the median (*P*_interaction_ < 0.001). Similarly, a cross-sectional study involving 250 pregnant women (65) observed significant interactions between fatty acid desaturase (*FADS*) SNPs (rs174561 and rs3834458) and dietary α-linolenic acid (ALA) and linoleic/α-linolenic acid ratio (LA/ALA) on plasma concentrations of omega-3 (n-3) PUFAs. It was reported that, in women with high ALA intake, plasma ALA concentrations were higher in homozygotes for the minor allele (*p* < 0.05), compared to carriers of the major allele (MM and Mm) of rs174561 and rs3834458. However, the *P*-values given in the study (*p* = 0.004 for rs174561 and *p* = 0.028 for rs3834458) seem to represent associations stratified by genotype, instead of interactions. FADS are involved in the synthesis of PUFA and their activation is linked to inflammation and coronary artery disease (70, 71), and these findings suggest that SNPs which alter the activation of FADS might affect plasma concentration of PUFA. In another cross-sectional study of 113 adolescents from the Obesity, Lifestyle and Diabetes in Brazil (BOLD) study (66), no significant interactions were reported between seven genes involved in the one-carbon metabolism pathway (Table 1) and fat intake on lipid-related traits.

#### 3.3.2. Interaction between dietary fat intake and genetic variants on glycemic traits

Interaction between dietary fat intake and genetic variants on glycemic traits was investigated by two cross-sectional studies (72, 73) using data from the BOLD study. In the first study which consisted of 200 participants (72), a high total fat intake [37.98% of total energy intake (TEI)/day] was shown to interact with a 10-SNP metabolic-GRS (Table 1), where individuals with 5 or more risk alleles had increased homeostasis model assessment estimate of insulin secretion (HOMA-B) (*P*_interaction_ = 0.016), fasting insulin (*P*_interaction_ = 0.017), body fat mass (*P*_interaction_ = 0.009), and decreased insulin:glucose ratio (*P*_interaction_ = 0.01), but the interaction did not influence homeostasis model assessment estimate of insulin resistance (HOMA-IR), glycated hemoglobin (HbA1c), or waist circumference (WC). Similarly, the second BOLD study (73) which also examined the interaction between dietary fat intake and a 10-SNP metabolic-GRS did not find significant interactions between the GRS and dietary fat intake on fasting glucose, fasting insulin or HbA1c (Table 1). The mechanisms through which dietary fat intake influence glycemic traits are unclear, although a sustained increase in blood glucose levels following a high fat meal has been reported (74).

#### 3.3.3. Interaction between plasma fatty acid profile and genetic variants on systemic inflammation

Five Brazilian cross-sectional studies (75–79) investigated the interaction between plasma fatty acids and genetic variants on systemic inflammation, using data from the HS-SP. The first study (75) consisted of 262 adults, and significant interactions were identified between plasma n-3 and adiponectin (*ADIPOQ*) SNP rs2241766 (*P*_interaction_ = 0.019); arachidonic acid and *ADIPOQ* rs16861209 (*P*_interaction_ = 0.044); docosapentaenoic acid and *ADIPOQ* rs16861209 (*P*_interaction_ = 0.037); and SFA and *ADIPOQ* rs17300539 (*P*_interaction_ = 0.019) on the risk of systemic inflammation. Carriers of the “G” allele of rs2241766 had a reduced odds ratio of having inflammatory biomarkers when plasma n-3 levels were above the median, while participants with the “CC” genotype of rs16861209 had a lower odds ratio of having inflammatory biomarkers in the 50th percentile of plasma arachidonic acid and docosapentaenoic acid. Moreover, carriers of the “A” allele of rs17300539 had a higher odds ratio of having inflammatory biomarkers in the upper 50th percentile of plasma SFA compared to those with the “GG” genotype (75). In the second study (76), which consisted of 262 participants, an interaction was also observed between plasma arachidonic acid/eicosapentaenoic acid ratio and toll-like receptor 4 (*TLR4*) SNP rs11536889, in which individuals with the “C” allele had an increased odds ratio of having inflammatory biomarkers at the higher percentile of arachidonic acid/eicosapentaenoic acid ratio (*P*_interaction_ = 0.034). Similarly, the third study consisting of 262 participants (77) identified a significant interaction between plasma palmitoleic acid and C-reactive protein (*CRP*) SNP rs1417938, where individuals with the “AA” genotype had a higher odds ratio of having inflammatory biomarkers with a plasma palmitoleic acid above the median (*P*_interaction_ = 0.047).

In line with these findings, an increasing risk of having inflammatory biomarkers in response to increasing plasma SFA was observed in carriers of the “A” allele of tumor necrosis factor-α (*TNF*-α) SNP rs180062 (−308G/A) (*P*_interaction_ = 0.041); while a decreasing risk with increasing plasma stearic acid was found in participants with the “GG” genotype (*P*_interaction_ = 0.046), in a sample of 281 participants from the HS-SP (78). Furthermore, a decreasing risk of metabolic syndrome (MetS) was observed in response to increasing plasma stearic acid levels in “A” allele carriers of interleukin 1 beta (*IL1B*) SNP rs16944 (*P*_interaction_ = 0.043), and in response to increasing plasma arachidonic acid levels in those with the “GG” genotype of interleukin 10 (*IL10*) SNP rs1800896 (*P*_interaction_ = 0.007), in a sample of 301 participants from the HS-SP (79). However, no significant interactions were identified between total SFA, myristic acid, palmitic acid, stearic acid and *ADIPOQ* SNPs rs1501299 and rs266729; *TLR4* SNPs rs11536889 and rs5030728; and *CRP* SNP rs1205 on inflammatory biomarkers in three of the studies (75, 78, 79). Plasma fatty acid profile is considered an indicator of dietary fatty acid intake (75) and these findings suggest that plasma fatty acid profile can interact with SNPs of several genes and modify the risk of systemic inflammation which is linked to cardiometabolic diseases such as type 2 diabetes and CVDs (75).

#### 3.3.4. Interaction between carbohydrate intake and genetic variants on cardiometabolic traits

Three Brazilian cross-sectional studies (66, 72, 73) investigated the interactions between carbohydrate intake and genetic variants on cardiometabolic traits, using data from the BOLD study. In the first study which consisted of 113 participants (66), a total carbohydrate intake of 47.7% TEI was associated with a significantly increased homocysteine concentration (*P*_interaction_ = 0.031) in carriers of the “*AA*” genotype of fucosyltransferase 2 (*FUT2*) SNP rs602662. Carbohydrate intake also interacted with Catechol-O-Methyltransferase (*COMT*) SNP rs4680, increasing oxidized-LDL more in carriers of “AA” than “GG” genotype (*P*_interaction_ = 0.005) (66). Notwithstanding, after applying Bonferroni correction for multiple testing, none of the interactions were considered significant (66). Moreover, the other two studies (72) which consisted of 200 participants and (73) which consisted of 187 participants, from the BOLD study, did not identify significant interactions between carbohydrate intake and a metabolic-GRS based on 10 SNPs (Table 1) on cardiometabolic traits.

#### 3.3.5. Interaction between protein intake and genetic variants on cardiometabolic traits

Three studies (66, 73, 80) investigated the interaction between protein intake and genetic variants on cardiometabolic traits, two of which (66, 73) used data from the BOLD study. A cross-sectional study of 1191 overweight and normal weight children (80) observed a significantly increased BMI (*p* = 0.01) among participants carrying the “*T*” allele of *FTO* SNP rs79149291 with a protein intake above 12.7% TEI/day (80). Similarly, in the BOLD study discussed above (66), those with a protein intake of 16.99% TEI who were carriers of the “*AA*” genotype of *FUT2* SNP rs602662 (*P*_interaction_ = 0.007) had increased homocysteine levels (66). However, in the other BOLD study (73), there were no interactions between protein intake and a GRS based on 10 SNPs (Table 1) on obesity or diabetes traits.

#### 3.3.6. Interactions between micronutrients and genetic variants on cardiometabolic traits

The interaction between micronutrients and genetic variants on cardiometabolic traits was examined by five Brazilian studies (81–85). A cross-sectional study of 335 healthy young adults (81), observed a pronounced increase in systolic blood pressure (SBP) (*P*_interaction_ = 0.016) among carriers of the “*G*” allele of Angiotensinogen (*AGT*) SNP rs699 with a higher plasma magnesium (209.3 mg). Similarly, among those with a high calcium intake (573.3 mg), carriers of the “*T*” allele of Bradykinin Receptor B2 (*BDKRB2*) SNP rs1799722 had significantly higher SBP (*P*_interaction_ = 0.015) and diastolic BP (DBP) (*P*_interaction_ = 0.014) than carriers of the “*CC*” genotype (81). In line with these findings, a case-control study of 234 elderly people (82) reported an interaction between sodium intake and angiotensin-converting enzyme (*ACE*) SNP rs4646994 on the risk of hypertension, where carriers of the “*I/I*” genotype with a high sodium intake (>2 g/day) had an increased risk of hypertension (*P*_interaction_ = 0.007). Furthermore, in a cross-sectional study of 1298 healthy adults (83), those carrying the “*T*” allele of Cytochrome B-245 Alpha Chain (*CYBA*) (*p22phox*) with more than 86.5 mEq sodium per 12 h of urine collection, had increased SBP (*P*_interaction_ < 0.001) and DBP (*P*_interaction_ = 0.011). Sodium is known to increase BP by reducing vasodilation (86), while dietary calcium is believed to stabilize intracellular calcium in smooth muscles, thereby reducing vasoconstriction and BP (87). Additionally, the “*A*” allele of *AGT* SNP rs699 is thought to be a risk factor for elevated SBP, possibly due to its association with a rise in plasma AGT levels (60, 81), and the findings of the study discussed above (81) indicate that, the protective effect of the “*G*” allele might be lost in the presence of higher plasma magnesium.

Similarly, in a longitudinal study of 1088 children with a follow up of 4.6 years (84), those with a deficit of plasma vitamin D (<75 nmol/L) and carriers of the risk allele (“*A*”) of *FTO* SNP rs9939609 had increased BMI (*P*_interaction_ = 0.033). However, a cross-sectional study examining folate intake in 5914 healthy adults (85) did not identify interactions between folate intake and *MTHFR* SNP rs1801133 on homocysteine concentrations.

#### 3.3.7. Interactions between alcohol intake and genetic variants on cardiometabolic traits

Three Brazilian studies (85, 88, 89) examined the interaction between alcohol intake and genetic variants on cardiometabolic traits. In a cross-sectional study of 113 participants (88), a significant interaction was observed between alcohol intake and endothelial nitric oxide synthase (*eNOS*) SNP rs2070744 (−786 T/C) on plasma nitrite levels. Individuals carrying the “C” allele who consumed alcohol had lower plasma nitrite levels (*P*_interaction_ = 0.033). However, there were no significant interactions between alcohol intake and rs2070744 on BP (88). Similarly, in a cross-sectional study of 3,803 participants from the Pelotas Birth Cohort (85), an interaction was identified between alcohol intake and *MTHFR* SNP rs1801133 (C677T), in which men with the “677TT” genotype who consumed ≥ 15 g of alcohol per day had the highest homocysteine concentration (*P*_interaction_ = 0.002); but the interaction was not observed in women. Moreover, a prospective cohort study of 964 postmenopausal women (89), reported no interactions between alcohol intake and *APOE* genotype on lipid traits. A rise in homocysteine concentration is attributed to a deficiency in B vitamins and folate, and SNPs of *MTHFR* might affect homocysteine concentration by impairing folate metabolism (90). However, it is unclear how alcohol intake modifies the activity of MTHFR, and the finding of the study (85) suggests a sex-specific response.

#### 3.3.8. Interactions between smoking and genetic variants on cardiometabolic traits

Two studies (91, 92) investigated the interaction between smoking and genetic variants on cardiometabolic traits in Brazilians. In a cross-sectional study of 391 participants (91), smoking interacted with *APOA-IV* SNPs rs693 (*Xba*I), rs675 (Thr347Ser) and rs5110 (Gln360His), increasing BMI in individuals with the “X*2” (*P*_interaction_ = 0.007) and “347Ser” (*P*_interaction_ = 0.02) alleles. However, men with the “360His” allele who were non-smokers had a larger WC than homozygotes for the “Gln” allele (*P*_interaction_ = 0.018) (91). Similarly, in a cross-sectional study of 673 overweight adults (403 women and 270 men) (92), carriers of the “S2” allele of *APOC3* SNP rs5128 had increased triglycerides and the effect was more pronounced in women who smoked than in non-smokers (*P*_interaction_ = 0.009). Serum APOC3 concentration has been shown to be positively associated with triglyceride levels, and smoking has been reported to lower the concentration of APOC3 but only in women without central obesity (93), indicating a sex-specific response which is influenced by obesity traits.

#### 3.3.9. Interactions between physical activity and genetic variants on cardiometabolic traits

Interactions between physical activity and genetic variants on cardiometabolic traits were investigated by nine Brazilian studies (66, 85, 91, 94–99). In a longitudinal study of 197 overweight or obese children (94), a physical exercise program (3 sessions/week for 12 weeks) interacted with adrenoceptor beta 2 (*ADRB2*) SNP rs1042714, decreasing triglyceride levels and triglyceride-glucose index (*P*_interaction_ = 0.001 for both) more in carriers of the “Glu27Glu” genotype than those carrying the “Gln27” allele. A cross-sectional study of 1701 children and adolescents (95) also reported higher BMI and WC in individuals with the “TT” genotype of fibronectin type III domain containing 5 (*FNDC5*) SNP rs16835198 compared to carriers of the “G” allele only in those with lower levels of cardiorespiratory fitness (CRF) (*P*_interaction_ = 0.038 and *P*_interaction_ = 0.007 for WC and BMI, respectively); and lower limb strength (*P*_interaction_ = 0.040 and *P*_interaction_ = 0.044 for WC and BMI, respectively). Physical activity has been proposed to alter the expression of certain genes (100), and the findings of these studies indicate that, the effect of physical activity on lipid, glycemic and anthropometric traits might be influenced by SNPs of *ADRB2* and *FNDC5* genes.

Similarly, a sedentary behavior (a screen time of > 378 min/day) was shown to increase cardiometabolic risk score in carriers of “AA” genotype of *FTO* SNP rs9939609 with a low CRF but not in those with a high CRF in a cross-sectional study of 1,215 children and adolescents (*P*_interaction_ = 0.047) (96). Along this line, a randomized controlled trial of 34 participants (97) reported that, a 45-min walk on a treadmill at moderate intensity resulted in a reduction in SBP (*P*_interaction_ = 0.02) and DBP (*P*_interaction_ < 0.01) in carriers of the “I” allele of *ACE* SNP rs4646994 compared with a non-exercise control session, but the reduction was not observed in participants with “DD” genotype. However, five studies (66, 85, 91, 98, 99) did not identify significant interactions between physical activity and genetic variants on cardiometabolic traits as shown in Table 1.

#### 3.3.10. Other gene-diet interactions in Brazilians

In the BOLD study consisting of 113 participants (66), a total fat intake of 25.36% TEI interacted with Betaine-Homocysteine S-Methyltransferase (*BHMT*) SNP rs492842, increasing vitamin B12 concentrations (*P*_interaction_ = 0.034) in participants with the “TT” genotype. A case-control interventional study of 126 obese women (101) also reported that, a hypocaloric diet (< 600 kcal/day) for 7 weeks was associated with a decreased abdominal circumference (*P*_interaction_ = 0.04) among carriers of the “*A*” allele of *FTO* SNP rs9939609. Furthermore, in a prospective cohort study of 3,701 women, breastfeeding (> 6 months duration) interacted with *FTO* SNP rs9939609, decreasing BMI (*P*_interaction_ = 0.03), fat mass (*P*_*interactin*_ = 0.03), and WC (*P*_interaction_ = 0.04) in carriers of the “*A*” allele (102).

In summary, research in Brazil stands out in comparison to the rest of the gene-lifestyle research in LACP for being the most abundant; twenty-nine studies investigated gene x lifestyle interactions in the Brazilian population as shown in Table 1, covering a wide range of cardiometabolic traits. Dietary fat intake and plasma fatty acid profile were the most frequently investigated dietary factors examined by seven and five studies, respectively, although all the studies examining plasma fatty acid profile used data from the HS-SP. Carbohydrate intake was examined by only three studies, all of which used data from the BOLD study. Similarly, protein intake was investigated by only three studies, two of which used data from the BOLD study. Physical activity was the most frequently examined lifestyle factor, followed by smoking and alcohol intake. Breastfeeding was examined by only one study (102), and lifestyle factors such as socioeconomic status, level of education, and the effect of rural and urban environments were not investigated. Only one study was conducted in rural settings (88), but it was not focused on interaction of the rural environment with genetic variants. The *FTO* SNP *rs9939609* was the most studied, being explored by five studies (84, 85, 96, 98, 99). Overall, the findings call for further research into lifestyle factors such as socioeconomic status, level of education and the effect of rural and urban environments as well as other dietary factors such as fruit and vegetable intake.

### 3.4. Gene x lifestyle interaction in Mexicans

#### 3.4.1. Interaction between dietary fat intake and genetic variants on CVD traits

The interaction between dietary fat intake and genetic variants on CVD-related traits was examined by five Mexican studies (103–107). In a cross-sectional study of 224 participants with T2D (103), interactions between monounsaturated fatty acid (MUFA) intake and *APOE* genotype on blood lipid concentrations were reported. A low MUFA intake (< 10–15% TEI) was found to be associated with higher total cholesterol (TC) (*P*_interaction_ = 0.016), non-HDL (*P*_interaction_ = 0.024) and LDL (*P*_interaction_ = 0.030) only in carriers of the “ε2” allele of *APOE* SNP rs7412. Similarly, interactions between MUFA intake (*P*_interaction_ = 0.001), total fat intake (*P*_interaction_ = 0.001), dietary cholesterol intake (*P*_interaction_ = 0.019) and Dopamine Receptor D2/Ankyrin Repeat and Kinase Domain Containing 1 (*DRD2/ANKK1*) SNP rs1800497, increasing triglyceride levels in carriers of the “A2A2” genotype were observed in a cross-sectional study of 175 Mexican adults with T2D (104). MUFA intake has been linked to decreased triglyceride concentration (108) which is consistent with the findings of the first study (103). However, the findings of the second study (104) imply that MUFA intake might not be beneficial for individuals with the “A2A2” genotype of rs1800497. Both studies were conducted in participants with T2D which is known to affect lipid metabolism (109). Moreover, as highlighted by the authors of the second study (104), the effect of dietary fat intake on triglycerides concentration may be influenced by other factors including physical activity and the level of insulin resistance.

A Mexican case-control study consisting of 100 participants with normal weight and 100 participants with obesity (105) also found significant interactions between SFA intake and leptin receptor (*LEPR*) SNP rs1137101 on TC (*P*_interaction_ = 0.002) and triglyceride (*P*_interaction_ = 0.02) levels. It was reported that, a SFA intake of ≥ 12 g/day was associated with a 3.8 times higher risk of hypercholesteroleamia and a 2.4 times higher risk of hypertriglycerideamia compared to an intake of < 12 g/day in participants carrying the “G” allele of rs1137101 (105). An interaction between total fat intake with *LEPR* SNP rs1137101 on TC (*P*_interaction_ = 0.001) was also reported in this study (105), where a high intake of total fat (≥83 g/d) was associated with a 4.1 times higher risk of hypercholesteroleamia in carriers of the “G” allele of rs1137101. Similarly, in a prospective cohort study involving a dietary intervention in 41 participants with hypercholesterolemia (106), interactions were observed between consumption of a diet low in SFA (<6% TEI/day) in addition to another diet containing 15 g of soluble fiber and 25 g of soy protein for 2 months and Glucose-Fructose Oxidoreductase Domain Containing 2 (*GFOD2*) SNP rs12449157 on TC (*P*_interaction_ = 0.006) and LDL (*P*_interaction_ = 0.025). Participants carrying the “G” allele had a larger decrease in TC and LDL in response to the dietary intervention compared to subjects with the “AA” genotype of rs12449157 (106). In this study (106), baseline LDL and TC levels were higher in carriers of the “G” allele, but they responded better to the dietary intervention, which indicates that the genetic risk of dyslipidaemia can be modified by a dietary intervention. However, in another study of 31 Mexican participants with dyslipidaemia (107) from the same cohort as above (106), using the same dietary intervention, no significant interactions were identified between the diet and Calpain 10 (*CAPN10*) SNPs rs5030952, rs2975762, and rs3792267 on lipid traits. It has been reported that SFA of different types and from different food sources might have different effects on cardiometabolic traits (110, 111), however, both studies (106, 107) used the same dietary intervention. Nonetheless, factors such as physical activity have also been reported to influence the effect of dietary fat intake on cardiometabolic traits (104), which could explain the differences in the findings.

#### 3.4.2. Interaction between carbohydrate intake and genetic variants on cardiometabolic traits

Interactions between carbohydrate intake and genetic variants on cardiometabolic traits were examined by three Mexican studies (104, 112, 113). In a cross-sectional study of 3591 adults (112), carbohydrate intake was negatively associated with HDL concentrations in premenopausal women carrying the risk allele (“C”) of ATP Binding Cassette Subfamily A Member 1 (*ABCA1*) SNP rs9282541 (*R230C*), but not in those carrying the “R” allele (*P*_interaction_ = 0.037). In another cross-sectional study of 215 healthy adults (113), a high sucrose intake (>5% TEI) significantly increased TC (*P*_interaction_ = 0.034) and LDL (*P*_interaction_ = 0.037) more in participants with “B1B2/B2B2” genotype than those with “B1B1” genotype of cholesteryl ester transfer protein (*CETP)* SNP rs708272. However, the interaction did not influence triglycerides, HDL, BMI nor waist circumference (113). In contrast, the cross-sectional study discussed above (104), reported that the intake of maltose (0.68 ± 0.42 g/day) significantly decreased triglycerides (P_interaction_ = 0.023) in carriers of the “A1”allele of *DRD2/ANKK1* SNP rs1800497. These findings indicate that carbohydrate intake might modulate lipid levels in Mexicans with certain genetic variants, but the mechanism through which carbohydrates affect lipid levels are unclear. Moreover, it has been reported that, the effect of carbohydrates on lipids might be dependent on glycemic index or glycemic load, and highly processed carbohydrates are linked to unfavorable lipid profiles (114).

#### 3.4.3. Interaction between micronutrients and genetic variants on cardiometabolic traits

Two cross-sectional studies examined the interaction between micronutrients and genetic variants on cardiometabolic traits (115, 116). In the first study which consisted of 231 healthy new-borns (115), a deficient maternal vitamin B12 (<2.0 mcg/d) was found to be associated with a smaller size baby at birth in mothers with the “TT” genotype of *MTHFR* SNP rs1801133 (*P*_interaction_ = 0.02) but a deficient maternal folate (<400 mcg/d) was not associated with anthropometric parameters (weight, length or BMI) of new-borns (115). A low vitamin B12 intake (<2.0 mcg/d) was also associated with increased homocysteine levels (*P*_interaction_ = 0.01) in carriers of the “TT” genotype of *MTHFR* SNP rs1801133 in a cross-sectional study of 130 healthy women (116). The “TT” genotype of *MTHFR* is associated with decreased enzymatic activity and increased homocysteine concentration (117) and the findings of these studies suggest that increasing the intake of vitamin B12 might improve fetal development in Mexican women with the “TT” genotype.

#### 3.4.4. Interaction between alcohol intake and genetic variants on cardiometabolic traits

The cross-sectional study of 130 healthy women discussed above (116), was the only study which examined alcohol intake and no interaction was found between alcohol intake and *MTHFR* SNP rs1801133 on homocysteine levels which could be due to the fact that 80% of the studied population consumed less than 1 cup/week of alcohol (116).

#### 3.4.5. Interaction between physical activity and genetic variants on cardiometabolic traits

Interactions between physical activity and genetic variants on cardiometabolic traits were investigated by four Mexican studies (113, 118–120). In the cross-sectional study discussed above (113), increased concentration of TC (*P*_interaction_ = 0.033) was observed in individuals carrying the “B2” allele of *CETP* SNP rs708272 who did not perform physical activity, compared to those with the “B1B1” genotype. However, there were no interactions on TG, HDL, TG:HDL ratio, LDL, BMI or WC (113). Similarly, interactions between physical fitness measured by muscular endurance (ME) and aerobic capacity with genetic variants were observed in a case-control study of 608 physically active adults (118), where higher levels of ME and aerobic capacity were associated with a lower WC in individuals with a high GRS based on 23 SNPs (Table 1) (*P*_interaction_ = 0.0001 for both). In this study (118), a higher risk of obesity was found in older participants (≥ 40 years) with the “AA” genotypes of *APOB* SNP rs512535 (P_interaction_ = 0.004) and tumor necrosis factor (*TNFA*) SNP rs361525 (*P*_interaction_ = 0.007) with low levels of ME. However, another cross-sectional study of 565 physically active participants (119) did not find significant interactions between physical fitness and six SNPs (*ADIPOQ* rs2241766, *ACSL1* rs9997745, *LIPC* rs1800588, *PPARA* rs1800206, *PPARG* rs1801282 and *PPARGC1A* rs8192678) on glycemic traits. Moreover, the fourth cross-sectional study which consisted of 394 participants (120), did not identify interactions between physical activity and *ADIPOQ* SNP –11391G/A on adiponectin levels.

#### 3.4.6. Other gene-lifestyle interactions in Mexicans

In a cross-sectional study of 206 Mexican women (121), an interaction between polycyclic aromatic hydrocarbons (PAHs) and Paraoxonase 1 (*PON1*) SNP rs661 (Q192R) on serum asymmetric dimethylarginine (ADMA) was observed, where individuals carrying the “R” allele had higher ADMA levels compared to those with the “QQ” genotype in response to higher levels of urinary 1-hydroxypyrene (*P*_interaction_ = 0.02). Increased levels of ADMA (*p* < 0.01) and fatty acid-binding protein 4 (*p* < 0.001) were also identified in individuals with the “RR” genotype of *PON1* SNP rs661 with higher urinary arsenic levels (>45.0 μg/g of creatinine) in comparison with participants with the “QQ” genotype in a sample of 185 Mexican women (122). The mechanisms of the interaction may be shared in the case of exposure to PAHs as these are also involved in the generation of reactive oxygen species (123).

Overall, different cardiometabolic traits have been investigated in Mexico, where eleven out of fifteen studies found significant gene x lifestyle interactions (103–106, 112, 113, 115, 116, 118, 121, 122) as shown in Table 1. Dietary fat intake was the most frequently examined dietary factor, being investigated by five studies (103–107); followed by carbohydrate intake, which was examined by three studies (104, 112, 113). Physical activity was the most frequently examined lifestyle factor, while alcohol intake was investigated by only one study. Lifestyle factors such as smoking, socioeconomic status, level of education and the impact of rural and urban environments were not investigated. Moreover, dietary factors such as consumption of protein, complex carbohydrates, and fruits and vegetables have not been investigated, highlighting a need for further research.

### 3.5. Gene x lifestyle interaction in Costa Ricans

#### 3.5.1. Interactions between dietary fat intake and genetic variants on CVD-related traits

The interaction between dietary fat intake and genetic variants on CVD-related traits was examined by six Costa Rican studies (124–129). In a cross-sectional study of 420 participants (124), SFA intake interacted with *APOE* genotype and influenced blood lipid concentrations. A higher SFA intake (13.5% energy) was associated with higher levels of very-low density lipoprotein cholesterol (VLDL) (*P*_interaction_ = 0.03) and lower concentration of HDL (*P*_interaction_ = 0.02) in carriers of the “ε*2*” allele. However, no significant interactions were identified between SFA intake and *APOE* genotype on lipids in a case-control study involving 1,927 participants with myocardial infarction (MI) and 1,927 matched controls (125). In another cross-sectional study of 336 participants (126), SFA intake was found to interact with *APOC3* genotype and impact on the concentration of TC (P_interaction_ = 0.0004) and LDL (P_interaction_ = 0.01). Homozygotes for the *APOC3-455T-625T* alleles had a 13% increase in TC and a 20% increase in LDL with a high SFA intake (>11% of energy intake), but the interaction was not significant in individuals with the *APOC3-455C-625del* allele (126). In the case-control study discussed above (125), a significant interaction between SFA intake and *APOE* genotype on the risk of MI (*P*_interaction_ = 0.0157) was also reported, in which carriers of the “ε*4”* allele had a 49% increased risk of MI compared to a 2.2 fold increased risk in those with the “ε*2”* allele in response to a high SFA intake (>11.8% of energy intake).

APOE plays a key role in lipid metabolism, being a main component of triglyceride-rich lipoproteins and HDL, and a ligand for LDL receptor (124, 130) and it is believed that the metabolism of fatty acids is impaired in carriers of the “ε*4*” allele which is considered a risk factor for CVDs (131). However, the above findings indicate that, a high SFA intake is more detrimental to carriers of the “ε*2*” allele than those carrying the “ε*4*” allele, highlighting the potential role of SFA intake in modifying genetic risk.

In accordance with the findings above, a case-control study of 1805 participants with a first non-fatal MI and 1,805 matched controls (127), reported an interaction between PUFA intake and *PPARγ* SNP rs1801282, influencing the risk of MI (*P*_interaction_ = 0.03). Individuals with the “Pro12/Pro12” genotype had a 34% reduced risk of MI per 5% increment in energy from PUFA compared to a 7% decreased risk in those carrying the “*Ala12*” allele (127). Similarly, a case-control study of 1932 participants with a first non-fatal MI and 2,055 matched controls (128), reported a significant interaction between long-chain omega-3 (LC n-3) PUFA intake and Proprotein Convertase Subtilisin/Kexin Type 9 (*PCSK9*) SNP rs11206510 on the risk of MI (*P*_interaction_ = 0.012), where carriers of the “C” allele had an odds ratio for MI of 0.84 per 0.1% increase in total energy from LC n-3 PUFA, compared to an odds ratio of 1.02 in participants without the “C” allele (128). Along similar lines, a case-control study of 1936 participants with a first non-fatal MI and 2,035 matched controls (129) reported a significant interaction between omega-6 (n-6) PUFA intake and Phospholipase A2 Group IVA (*PLA2G4A*) SNP rs12746200 on the risk of MI (*P*_interaction_ = 0.005), in which participants with the “G” allele had a reduced risk of MI with an intake of n-6 PUFA above the median compared to those with the “AA” genotype. However, there were no significant interactions with n-3 PUFA intake (129).

These findings indicate that the beneficial effect of PUFA intake reported by some studies (101, 132) might be limited in individuals with certain genetic variants. PPARγ is a nuclear receptor which is involved in adipogenesis and plays a role in the metabolism of glucose and fatty acids (133, 134), and the “*Ala12*” allele of PPARγ SNP rs1801282 has been reported to slow down the release of PUFA from adipocytes, which could explain the smaller reduction in the risk of MI in comparison with carriers of the “Pro12/Pro12” genotype (127).

#### 3.5.2. Interaction between other dietary factors and genetic variants on the risk of MI

Interactions between other dietary factors and genetic variants on the risk of MI were examined by three Costa Rican studies (135–137). In a case-control study of 1,560 incident cases of non-fatal MI and 1,751 matched controls (135), sugar sweetened beverage (SSB) intake interacted with a GRS based on 3 SNPs of chromosome 9p21 (rs4977574, rs2383206 and rs1333049), increasing the risk of MI (*P*_interaction_ = 0.03). SSB intake also interacted with rs4977574, increasing the risk of MI in carriers of the “G” allele (*P*_interaction_ = 0.005), but there was no interaction with fruit juice intake (135). In another case-control study of 2,014 participants with a first acute non-fatal MI and 2,014 matched controls (136), an increased risk of MI with increasing coffee intake was observed in carriers of the “C” allele (also known as “slow metabolizers of caffeine”) of Cytochrome P450 Family 1 Subfamily A Member 2 (*CYP1A2*) SNP rs762551 compared to carriers of the “AA” genotype (*P*_interaction_ = 0.04). Similarly, in a case-control study consisting of 2,042 participants with a first non-fatal MI and 2042 control subjects (137), cruciferous vegetable intake (0.86 servings/day of half a cup) interacted with Glutathione S-Transferase Theta 1 (*GSTT1*) SNP rs17856199, lowering the risk of MI in carriers of the “*1” allele, but not in individuals with the “*0*0” genotype (*P*_interaction_ = 0.006). These findings indicate that, dietary factors other than fat intake, might also influence the risk of MI in Costa-Ricans with certain genetic variants.

#### 3.5.3. Interaction between smoking and genetic variants on the risk of MI

Interaction between smoking and genetic variants on the risk of MI was investigated by three Costa Rican case-control studies (137–139), two of which found significant interactions (137, 138). In a case-control study of 492 participants with a first non-fatal MI and 518 matched controls (138), an interaction was observed between smoking status and Paraoxonase 1 (*PON1*_192_) SNP rs661 on the risk of MI (*P*_interaction_ = 0.04), where the *PON1*_192A*rg*_ allele was associated with an increased risk of MI only in non-smokers. Similarly, in the case-control study discussed above (137), the combined intake of cruciferous vegetables (>5 servings/day) and smoking (1–10 cigarettes/day) in carriers of the “*1” allele of rs17856199, lowered the risk of MI (*P*_interaction_ = 0.008). However, there were no significant interactions with *GSTM1* or *GSTP1* genotype on the risk of MI (137). Moreover, in the third Costa Rican case-control study which involved 873 participants with a first non-fatal MI and 932 control subjects (139), no significant interactions were observed between smoking and *CYP1A1* SNP rs1048943 or *CYP1A2* SNP rs762551 on the risk of MI. Smoking has been linked to increased risk of MI (140, 141) although the mechanisms are unclear. Smoking is also believed to impair the activity of PON1, which is linked to increased risk of CVDs (142, 143), but this is not supported by the findings of the studies above, suggesting that Costa Ricans with certain genetic variants might respond differently to smoking.

#### 3.5.4. Other gene-lifestyle interactions in Costa Ricans

One case-control study consisting of 1534 participants with a first non-fatal MI and 1,534 matched controls (144), investigated the interaction between a lifestyle cardiovascular risk score comprising of physical activity, smoking, alcohol consumption, waist-to-hip ratio, and socioeconomic status; and a GRS based on 14 SNPs (Table 1) on the risk of MI, and no significant interactions were identified.

The research in Costa Rica has mainly focused on CVD traits in adults, with an emphasis on the risk of MI, and dietary fat intake has been the most frequently examined exposure. Socioeconomic status was examined by one study (144), and lifestyle factors such as educational level, the effect of rural and urban environments as well as dietary factors such as consumption of protein, fiber and complex carbohydrates have not been explored, highlighting a need for further research.

### 3.6. Gene x lifestyle interaction in LACP diaspora

#### 3.6.1. Interaction between dietary fat intake and genetic variants on anthropometric traits

Interaction between dietary fat intake and genetic variants on anthropometric traits were investigated by six studies (145–150), all of which used data from the Boston Puerto Rican Health Study (BPRHS). In a cross-sectional study of 930 Puerto Ricans from the BPRHS (145), a high intake of SFA (≥22 g/day) was associated with a 7.9% higher BMI in individuals with the “CC” genotype of *APOA2* SNP rs5082 than those carrying the “T” allele (*P*_interaction_ = 0.003); but the SNP had no effect on BMI when SFA intake was low (<22 g/day). This study also observed that, among individuals with a high SFA intake (≥22 g/d), those with the “CC” genotype had a higher risk of obesity than participants carrying the “T” allele of the SNP rs5082 [Odds ratio (OR) = 1.84; 95% confidence interval (CI) = 1.38–2.47; *P* < 0.0001). A similar finding was reported in a prospective cohort study of 920 participants from the BPRHS (146), where a high intake of SFA (≥ 9.3% of total energy) was linked to higher BMI (*P*_interaction_ = 0.006), WC (*P*_interaction_ = 0.02), and hip circumference (HC) (*P*_interaction_ = 0.002) in participants carrying the minor allele (“T”) of LDL receptor related protein 1 (*LRP1*) SNP rs1799986 compared to individuals with the “CC” genotype; but the SNP had no effect on anthropometric traits when SFA intake was low (<9.3% of total energy). The “CC” genotype of *APOA2* rs5082 is believed to affect body fat distribution by lowering plasma concentration of APOA2 and these findings indicate that, a low SFA intake might attenuate this genetic risk (145, 151).

An interaction of total fat intake with *APOA1*-75 on WC was also reported in a longitudinal study of 821 participants of the BPRHS (147), in which individuals carrying two copies of the major allele had a lower WC with a low total fat intake than those carrying the minor allele (P_interaction_ = 0.005). A longitudinal study performed in 1,171 participants (333 men and 838 women) of the BPRHS (148) also observed that, women with the “TT” genotype of lipoprotein lipase (*LPL)* SNP rs320 had lower BMI (*P*_interaction_ = 0.002) and WC (P_interaction_ = 0.001) with a high intake of PUFA but this was not observed in minor allele (“G”) carriers and there were no significant interactions in men. In contrast, another longitudinal study of 1,340 participants (395 men and 945 women) of the BPRHS (149) found that, men with the “GG” genotype of brain derived neurotrophic factor (*BDNF)* SNP rs6265 had higher BMI (*P*_interaction_ = 0.042), WC (*P*_interaction_ = 0.018), and HC (*P*_interaction_ = 0.009) with a low PUFA intake (<8.76% of energy) than those carrying the “A” allele but no difference was observed when PUFA intake was high (≥8.76% of energy) and the interaction was not observed in women. Interaction between Mediterranean diet with *TCF7L2* SNP rs7903146 on obesity-related traits was also observed in a cross-section study of 1,120 Puerto Ricans of the BPRHS (150), where carriers of the “T” allele had lower WC (99.2 ± 0.9 vs. 102.2 ± 0.9 cm; *P*_interaction_ = 0.026) and weight (77.3 ± 1.0 vs. 80.9 ± 1.0 kg; *P*_interaction_ = 0.024) with a high Mediterranean diet score than individuals with “CC” genotype. However, there were no significant differences between the genotypes when the Mediterranean diet score was low. The findings suggest that a high intake of PUFA and Mediterranean diet might be beneficial in reducing the genetic risk of obesity-related traits in a sex-specific manner and call for further research into the mechanisms involved.

#### 3.6.2. Interaction between dietary fat intake and genetic variants on CVD traits

Interaction between total fat intake and genetic variants on CVD traits were reported by three studies (147, 152, 153). In a longitudinal study of 802 participants of the BPRHS (152), a significant interaction was observed between total fat intake and *APOA5* SNP -1131T < C on plasma triglycerides (P_interaction_ = 0.032), where a high total fat intake (≥31% of total energy) was associated with a higher plasma triglyceride concentration in individuals with the “1131C” allele, although no difference between the genotypes was observed when total fat intake was low. This study (152) also observed an interaction between *APOA5* SNP S19W with total fat intake on SBP (*P*_interaction_ = 0.002) and DBP (*P*_interaction_ = 0.007), where participants with the minor allele (“G”) had a higher SBP with a low total fat intake (< 31% of total energy), and a lower SBP with a high total fat intake in comparison with individuals with the “CC” genotype. The study on 821 participants of the BPRHS discussed above (147), also reported significant interactions between total fat intake and *APOC3* -640 on DBP (*P*_interaction_ = 0.003), *APOA4* N147S and *APOA5* S19W on SBP (*P*_interaction_ = 0.001 and *P*_interaction =_ 0.002, respectively). It was observed that, homozygous for the major allele of *APOA1*-75, *APOA4* N147S and *APOA5* S19W had lower SBP with a low intake of total fat (< 31% of total energy) than those carrying the minor allele; while heterozygous for *APOC3* -640 had lower DBP with a high total fat intake (≥ 31% from energy) (147). However, a randomized crossover trial involving 41 adults from Dominican, Puerto Rican and other Caribbean Hispanic origins (153), did not find significant interactions between a high fat diet and hepatic lipase (*LIPC*) SNP rs1800588 on HDL, LDL, TC or plasma glucose concentrations. A high intake of total fat has been associated with an unfavorable lipid profile and high blood pressure (154) and the above findings indicate that, this association might be influenced by variants of several genes.

#### 3.6.3. Interaction between carbohydrate intake and genetic variants on cardiometabolic traits

Two studies investigated the interaction between carbohydrate intake and genetic variants on cardiometabolic traits (155, 156). In a longitudinal study involving 920 participants of the BPRHS (155), a significant interaction was observed between Perilipin 1 (*PLIN 1*) SNP 1,482 G > A and complex carbohydrate intake on WC (*P*_interaction_ = 0.002), where individuals carrying the “A” allele had a higher WC with a low intake of complex carbohydrate (<144 g/day) and a lower WC with a high intake of complex carbohydrate (≥144 g/day) than those with the “GG” genotype. Similarly, a cross-sectional study of 153 children descendent from Hispanic ancestry (156), identified significant interaction between carbohydrate intake (211.4 g/day) and total sugar intake (96.1 g/day), increasing hepatic fat fraction in carriers of the “GG” genotype of Patatin like phospholipase domain containing 3 (*PNPLA3*) SNP rs738409 (*P*_interaction_ = 0.04 and *P*_interaction_ = 0.01, respectively), but the interaction was not observed in individuals carrying the “C” allele. It has been reported that, body weight might be influenced by the type of carbohydrate consumed (157) which is supported by the findings of these studies, but the results also indicate that genetic variants might also play a role.

#### 3.6.4. Interaction between micronutrient intake and genetic variants on cardiometabolic traits

The interaction between micronutrient intake and genetic variants on cardiometabolic traits was investigated by two studies (158, 159). A cross-sectional study involving 1,734 Mexican Americans (158) reported a significant interaction between vitamin E and *APOB* SNP rs693 on LDL (*P*_interaction_ = 8.94 × 10-7), and between vitamin A and *PCSK9* SNP rs11206510 on LDL (*P*_interaction_ = 7.65 × 10-5), but the direction of the interactions is unclear. Similarly, in the longitudinal study of 1,144 Puerto Ricans of the BPRHS discussed above (159), a significant interaction between vitamin D status and *IRS1* rs2943641 on the risk of T2D was identified in women in which minor allele homozygotes (“TT”) had a lower risk of T2D compared with “C” allele carriers only when 25(OH)D was higher than the median [>17 ng/mL (42.4 nmol/L)] (*P*_interaction_ = 0.007), but the interaction was not observed in men. The findings of these studies indicate that micronutrients might modulate the association between genetic variants and lipid and glycemic traits, but further studies are needed to replicate and elucidate the mechanisms involved.

#### 3.6.5. Interaction between physical activity and genetic variants on cardiometabolic traits

Only one study (160) examined the interaction between physical activity and genetic variants on cardiometabolic traits. This study (160) was a prospective cohort study of 9,645 adult Puerto Ricans, Mexicans, Dominicans, Cuban, Central American, and South American from the Hispanic Community Health Study/Study of Latinos (HCHS/SOL) cohort, USA, and a positive association was observed between a GRS based on 97 SNPs (Table 1) and BMI, but the effect of the GRS was stronger in the first tertile of moderate to vigorous physical activity compared to the third tertile (*P*_interaction_ = 0.005). Significant interactions following the same pattern were observed for fat mass (*P*_interaction_ = 0.003), fat percentage (*P*_interaction_ = 0.003) and fat mass index (*P*_interaction_ = 0.002) (160).

In summary, research in LACP diaspora has mainly focused on Puerto Ricans residing in USA and most of this evidence (10 out of 13 studies) comes from the same study (BPRHS). Dietary fat intake has been the most frequently studied, with carbohydrate intake being examined by only two studies. Similarly, physical activity was investigated by only one study and lifestyle factors such as socioeconomic status, level of education, and the effect of rural and urban environments have not been explored.

### 3.7. Gene x lifestyle interactions in Chileans

#### 3.7.1. Interaction between carbohydrate intake and genetic variants on glycemic traits

Two gene-diet interaction studies were reported in Chileans (161, 162). The first study (161) was a cross-sectional study of 2828 healthy Chilean adults, and a significant interaction was observed between consumption of SSB and a weighted genetic risk score (wGRS) based on 16 T2D risk SNPs (Table 1) on log-fasting glucose (*P*_interaction_ = 0.02), where the strongest effect was observed between the highest SSB intake (≥2 servings/day of 330 ml) and the highest wGRS. In this study (161), SSB intake also interacted with additive effects of Transcription Factor 7 Like 2 (*TCF7L2*) SNP rs7903146 (*P*_interaction_ = 0.002) and with the “G/G” genotype of Melatonin Receptor 1B (*MTNR1B*) SNP rs10830963 (*P*_interaction_ = 0.001), increasing log-fasting glucose levels. The second Chilean study (162) was a non-randomized controlled trial performed in 97 healthy women and 147 women with polycystic ovary syndrome, and there were no reported interactions between a high glycemic carbohydrate intake (75 g of glucose) during an oral glucose tolerance test and Insulin Receptor Substrate 1 (*IRS-1*) SNP rs1801278 on glycemic traits. In Chile, research has been limited to diabetes traits as outcomes and simple carbohydrates as exposure, reflecting a need for further research into other dietary and lifestyle factors such as socioeconomic status, level of education and the effect of rural and urban environments.

### 3.8. Gene x lifestyle interactions in Colombians

Two gene-lifestyle interaction studies were conducted in Colombians (163, 164). The first study (163) was a case-control study involving 212 normal weight, 112 overweight and 100 obese teenagers and no significant interactions were observed between physical activity and three SNPs (Uncoupling Protein 3 (*UCP3*) rs1800849, *FTO* rs17817449, and *CAPN10* rs3842570) on excess weight. However, sub-group analysis showed that, a sedentary lifestyle was associated with an increased risk of excess weight only in those with the “GG” or ‘TT’ genotype of *FTO* rs17817449 (*p* = 0.0005); and ‘CC’ genotype of *UCP3* rs1800849 (*p* = 0.0032) (163). It was also observed that, even with an active lifestyle [1.6–1.9 metabolic equivalent task (MET) minute/day], individuals with the “II” genotype of *CAPN10* rs3842570 had a higher risk of excess body weight compared to those carrying the “D” allele (*p* = 0.0212) (163). The second study which was also a cross-sectional study involved 1,081 Colombian teenagers (164), and there were no interactions between lifestyle factors (socioeconomic stratum, level of education and maternal breastfeeding) and ten SNPs on BMI (Table 1). As both studies (163, 164) were conducted in teenagers and focused on obesity traits, there is a need for further research into other cardiometabolic traits in the wider Colombian population.

### 3.9. Gene x lifestyle interactions in Argentinians

Only one study (165) was conducted in Argentinians, and this was a cross-sectional study consisting of 572 healthy Argentinian men. This study (165) reported a significant interaction between smoking status and *PPARγ* SNP rs1801282 on the risk of MetS (*P*_interaction_ = 0.031) where among the non-smokers, carriers of the “*Pro/Ala*” genotype (*p* = 0.0059) and the “*Ala12*” allele (*p* = 0.009) had a higher risk of MetS than non-carriers. It is unclear whether there were significant interactions between smoking status and rs1801282 genotype on the other outcomes investigated in the study (165) (Table 1), since the *p*-values given are for associations stratified by smoking status. The study adjusted for BMI and age only, but the pathophysiological mechanism of MetS is multifactorial (166), and hence other factors should be considered simultaneously. There have been no studies in Argentina examining the interactions of genetic variants with dietary factors, physical activity, or other lifestyle factors apart from smoking status.

## 4. Summary of the findings of commonly investigated interactions across the countries

The most commonly investigated interactions in LACP related to dietary fat intake and genetic variants on blood lipids. A high intake of olive oil was associated with lower LDL in Brazilian men with the “ε*2*” allele of *APOE* (62), while a low MUFA intake was linked to higher TC, non-HDL and LDL in Mexicans carrying the “ε*2*” allele of *APOE* (103). In contrast, increased TG concentration in response to a high MUFA intake was observed in Mexicans who were homozygotes for the A2 allele of *DRD2/ANKK1* SNP rs1800497. A high PUFA intake was also associated with increased concentration of LDL in Brazilian carriers of the “ε*4*” allele, and reduced concentration of TG in those carrying the “ε*2*” allele of *APOE* (62). However, a low PUFA intake was linked to increased TG and VLDL concentration in Brazilian women with the “ε*4*” allele of *APOE* (63).

Furthermore, a high SFA intake was associated with higher VLDL and lower HDL concentrations in Costa Rican carriers of the “ε*2*” allele of *APOE* (124), but no significant interactions were identified between SFA intake and *APOE* genotype on blood lipids in a Costa-Rican case-control study involving participants with MI (125). However, a high SFA intake was linked to increased concentrations of TC and LDL in Costa Ricans who were homozygotes for the *APOC3-455T-625T* alleles (126). Similarly, a high SFA intake was associated with increased TC and TG concentrations in Mexicans with the “G” allele of *LEPR* SNP rs1137101 (105); while a low SFA intake was linked to a decrease in TC and LDL concentrations in Mexicans with the “G” allele of *GFOD2* SNP rs12449157 (106).

The inconsistencies in the findings of the above studies call for further research into the interaction between sub-types of fat and genetic variants on blood lipids. The sources of dietary fat also need to be considered since SFA from different food sources have been reported to have different effects on cardiometabolic traits (111).

## 5. Discussion

This is the first systematic review to investigate gene-lifestyle interactions on cardiometabolic diseases in LACP, highlighting several gene-lifestyle interactions with effects being significant in Brazilians, Mexicans, Costa Ricans, Chileans, Argentinians, Colombians and LACP diaspora. The most frequently studied genes have been *FTO*, examined in Colombians, Mexicans, and Brazilians, *APOE* explored in Costa Ricans, Mexicans, and Brazilians, and *TCF7L2* investigated in Chileans, Mexicans, Brazilians and LACP diaspora. The concentration of blood lipids such as HDL and LDL was the most widely investigated trait, followed by BMI and WC; MI was examined by 11 studies and one study looked at hepatic fat accumulation, while diseases such as stroke and liver cirrhosis were not investigated. Research has identified gene-lifestyle interactions that describe effects which are population-, gender-, and ethnic-specific. The findings of this review indicate that most of the gene x lifestyle interactions were conducted once, necessitating replication to strengthen the evidence.

Another issue that could affect the results is the accuracy of the methods used to measure exposure variables such as dietary intake and physical activity (167, 168). Some studies used 24-h recall questionnaires and self-reporting methods (64, 77, 81, 112, 144, 158), which might have induced recall bias, inadequate estimations, daily variation bias, and over and underreporting of values (169, 170). Measurement of dietary intake is a crucial part of gene-diet interaction studies as under or overestimation of dietary intake can weaken or reverse the association between dietary factors and cardiometabolic traits (170, 171). Moreover, other studies used food frequency questionnaires with no information on whether they were tested for validity. Genotyping errors can also affect the results of gene-diet interactions by leading to deviations from the true genotype (172, 173).

Sample size has also been highlighted as a key methodological issue in gene-lifestyle interaction studies (167, 168). For complex traits where the main effects of genetic variants are often modest, a large sample size is required to detect small interaction effects (167, 174). Thus, it is important that studies are adequately powered to detect true interactions (168). Nonetheless, most of the studies had small sample sizes and only a few included information on statistical power to detect interactions. There is also the risk of false-positive finding when there is no correction for multiple comparisons (173, 175), but only a few of the studies provided information on correction for multiple comparisons.

Overall, the included studies are majorly cross-sectional, indicating a need for longitudinal/prospective studies. The findings reflect gaps in covering the genetic risks and the socioeconomic variables to which the LACP are exposed; 27 out of 33 LACP have not conducted gene-lifestyle interaction studies yet. Only five studies have been conducted in contexts of low socioeconomic status, and from these, only two studies investigated gene-socioeconomic status interactions (144, 164). Moreover, no studies have examined the impact of rural and urban environments on the genetic predisposition to cardiometabolic diseases, highlighting a gap in knowledge in LACP. The higher number of nutrigenetic studies in Brazil compared to the other countries could be attributed to several factors including existing data on genetic studies (176–181), GWAS done mainly in Brazil (182–184), increased awareness on nutrigenetics in Brazil or more research facilities available in Brazil compared to other LACP. Future gene-lifestyle interaction studies will need to replicate primary research of already studied genetic variants to enable comparison, and to explore the interactions between genetic and other lifestyle factors such as those conditioned by socioeconomic factors and the built environment. Moreover, the molecular mechanisms that underlie the gene-lifestyle interactions identified by this systematic review need to be explored. The strength of this review is the comprehensive search strategy and the inclusion of all dietary/lifestyle exposures and cardiometabolic traits. Another strength is the use of standardized tools to assess the quality of the studies. However, the study has some limitations.

In conclusion, this systematic review has identified several gene-lifestyle interactions on cardiometabolic disease traits in Brazilians, Mexicans, Costa Ricans, Chileans, Argentinians, Colombians and LACP diaspora, highlighting effects which are population-, gender-, and ethnic-specific. However, the lack of replication of most of the gene-lifestyle interactions made it difficult to evaluate the evidence. Moreover, most of the studies were cross-sectional meaning that they preclude causal assumptions hence a temporal relationship cannot be established. Future gene-lifestyle interaction studies will need to replicate primary research of already studied genetic variants to enable comparison, and to explore the interactions between genetic and other lifestyle factors such as those conditioned by socioeconomic factors and the built environment. Moreover, the molecular mechanisms that underlie the gene-lifestyle interactions identified by this systematic review need to be explored.

## Data availability statement

The original contributions presented in this study are included in the article/Supplementary material, further inquiries can be directed to the corresponding author.

## Author contributions

KV: conceptualization, supervision, and project administration. EV, KV, and RW: methodology, validation, investigation, writing—original draft preparation, and resources. EV and RW: software, formal analysis, data curation, and visualization. AS and KV: funding acquisition. All authors contributed to writing—review and editing and read and agreed to the final version of the manuscript.
