# Supplementary material for: Interactions between genetic and lifestyle factors on cardiometabolic disease-related outcomes in Latin American and Caribbean populations: A systematic review
Source: Front Nutr. 2023 Jan 26;10:1067033. doi: 10.3389/fnut.2023.1067033 (PMC9909204; doi:10.3389/fnut.2023.1067033)
Supplement: Supplementary file 1 [file Data_Sheet_1.docx]

Supplementary Material

# Section 1: Search strings

Table S1. Number of Hits and Search Strings per Database

| Search engine/database | Search string | Nº of HITS |
| --- | --- | --- |
| Web of Science | Genetic*(all fields) and interaction*(all fields) and diet* (all fields) and Latin (all fields) | 34 |
|  | Gene*(all fields) AND interact*(all fields) AND Caribbean (all fields) AND diet*(all fields) | 47 |
|  | Genetic*(all fields) AND interact*(all fields) AND latin*(all fields) AND physical*(all fields) | 25 |
|  | Gene*AND interact* AND caribbean AND physical* | 12 |
|  | gene (All fields) and interaction (All fields) and haiti (All fields) | 4 |
|  | gene (All fields) and interaction (All fields) and Cuba (All fields) | 126 |
|  | gene (All fields) and interaction (All fields) and Dominican (All fields) | 37 |
|  | gene (All fields) and interaction (All fields) and Jamaica (All fields) | 70 |
|  | gene (All fields) and interaction (All fields) and Trinidad (All fields) | 94 |
|  | gene (All fields) and interaction (All fields) and Bahamas (All fields) | 16 |
|  | gene (All fields) and interaction (All fields) and Barbados (All fields) | 17 |
|  | gene (All fields) and interaction (All fields) and Saint Lucia (All fields) | 574 |
|  | gene (All fields) and interaction (All fields) and Grenada (All fields) | 20 |
|  | gene (All fields) and interaction (All fields) and Grenadines (All fields) | 1 |
|  | gene (All fields) and interaction (All fields) and Antigua and Barbuda (All fields) | 0 |
|  | gene (All fields) and interaction (All fields) and Dominica (All fields) | 10 |
|  | gene (All fields) and interaction (All fields) and Saint Kitts & Nevis (All fields) | 0 |
|  | gene (All fields) and interaction (All fields) and Mexico (All fields) | 3,116 |
|  | gene (All fields) and interaction (All fields) and Guatemala (All fields) | 24 |
|  | gene (All fields) and interaction (All fields) and Honduras (All fields) | 19 |
|  | gene (All fields) and interaction (All fields) and Nicaragua (All fields) | 11 |
|  | gene (All fields) and interaction (All fields) and Salvador (All fields) | 405 |
|  | gene (All fields) and interaction (All fields) and costa rica (All fields) | 125 |
|  | gene (All fields) and interaction (All fields) and Panama (All fields) | 145 |
|  | gene (All fields) and interaction (All fields) and Belize (All fields) | 8 |
|  | gene (All fields) and interaction (All fields) and Brazil (All fields) | 5,344 |
|  | gene (All fields) and interaction (All fields) and colombia (All fields) | 571 |
|  | gene (All fields) and interaction (All fields) and argentina (All fields) | 1,621 |
|  | gene (All fields) and interaction (All fields) and peru (All fields) | 139 |
|  | gene (All fields) and interaction (All fields) and venezuela (All fields) | 131 |
|  | gene (All fields) and interaction (All fields) and chile (All fields) | 1,061 |
|  | gene (All fields) and interaction (All fields) and Ecuador (All fields) | 106 |
|  | gene (All fields) and interaction (All fields) and bolivia (All fields) | 29 |
|  | gene (All fields) and interaction (All fields) and paraguay (All fields) | 33 |
|  | gene (All fields) and interaction (All fields) and uruguay (All fields) | 229 |
|  | gene (All fields) and interaction (All fields) and guyana (All fields) | 8 |
|  | gene (All fields) and interaction (All fields) and suriname (All fields) | 2 |
| PubMed | (((genetic*) AND (interaction*)) AND (diet*)) AND (latin) | 45 |
|  | (gene-diet interaction) AND (latin*) | 8 |
|  | (((genetic*) AND (interaction*)) AND (nutrient)) AND (Caribbean) | 7 |
|  | (((genetic*) AND (interaction*)) AND (nutrient)) AND (latin) | 16 |
|  | (((genetic*) AND (interaction*)) AND (physical)) AND (latin) | 18 |
|  | (gene-nutrient interaction) AND (latin*) | 3 |
|  | "gene"[Title/Abstract] AND "interaction"[Title/Abstract] AND "Puerto Rico"[Title/Abstract] | 18 |
|  | "gene"[Title/Abstract] AND "interaction"[Title/Abstract] AND "Falkland"[Title/Abstract] | 0 |
|  | "gene"[Title/Abstract] AND "interaction"[Title/Abstract] AND "Montserrat"[Title/Abstract] | 0 |
|  | "gene"[Title/Abstract] AND "interaction"[Title/Abstract] AND "Anguilla"[Title/Abstract] | 15 |
|  | "gene"[Title/Abstract] AND "interaction"[Title/Abstract] AND "British Virgin Islands"[Title/Abstract] | 0 |
|  | "gene"[Title/Abstract] AND "interaction"[Title/Abstract] AND "Caicos"[Title/Abstract] | 1 |
|  | "gene"[Title/Abstract] AND "interaction"[Title/Abstract] AND "Cayman Islands"[Title/Abstract] | 0 |
|  | "gene"[Title/Abstract] AND "interaction"[Title/Abstract] AND "Caribbean Netherlands"[Title/Abstract] | 0 |
|  | "gene"[Title/Abstract] AND "interaction"[Title/Abstract] AND "Maarten"[Title/Abstract] | 0 |
|  | "gene"[Title/Abstract] AND "interaction"[Title/Abstract] AND "Aruba"[Title/Abstract] | 0 |
|  | "gene"[Title/Abstract] AND "interaction"[Title/Abstract] AND "Curaçao"[Title/Abstract] | 2 |
|  | "gene"[Title/Abstract] AND "interaction"[Title/Abstract] AND "Guiana"[Title/Abstract] | 3 |
|  | "gene"[Title/Abstract] AND "interaction"[Title/Abstract] AND "Martinique"[Title/Abstract] | 1 |
|  | "gene"[Title/Abstract] AND "interaction"[Title/Abstract] AND "Guadeloupe"[Title/Abstract] | 4 |
|  | "gene"[Title/Abstract] AND "interaction"[Title/Abstract] AND "Suriname"[Title/Abstract] | 0 |
|  | "gene"[Title/Abstract] AND "interaction"[Title/Abstract] AND "Guyana"[Title/Abstract] | 3 |
|  | "gene"[Title/Abstract] AND "interaction"[Title/Abstract] AND "Uruguay"[Title/Abstract] | 6 |
|  | "gene"[Title/Abstract] AND "interaction"[Title/Abstract] AND "Paraguay"[Title/Abstract] | 1 |
|  | "gene"[Title/Abstract] AND "interaction"[Title/Abstract] AND "Bolivia"[Title/Abstract] | 3 |
|  | "gene"[Title/Abstract] AND "interaction"[Title/Abstract] AND "Ecuador"[Title/Abstract] | 4 |
|  | "gene"[Title/Abstract] AND "interaction"[Title/Abstract] AND "Chile"[Title/Abstract] | 18 |
|  | "gene"[Title/Abstract] AND "interaction"[Title/Abstract] AND "Venezuela"[Title/Abstract] | 9 |
|  | "gene"[Title/Abstract] AND "interaction"[Title/Abstract] AND "Peru"[Title/Abstract] | 11 |
|  | "gene"[Title/Abstract] AND "interaction"[Title/Abstract] AND "Argentina"[Title/Abstract] | 19 |
|  | "gene"[Title/Abstract] AND "interaction"[Title/Abstract] AND "Colombia"[Title/Abstract] | 22 |
|  | "gene"[Title/Abstract] AND "interaction"[Title/Abstract] AND "Brazil"[Title/Abstract] | 126 |
|  | "gene"[Title/Abstract] AND "interaction"[Title/Abstract] AND "Belize"[Title/Abstract] | 2 |
|  | "gene"[Title/Abstract] AND "interaction"[Title/Abstract] AND "Panama"[Title/Abstract] | 7 |
|  | "gene"[Title/Abstract] AND "interaction"[Title/Abstract] AND "Costa Rica"[Title/Abstract] | 14 |
|  | "gene"[Title/Abstract] AND "interaction"[Title/Abstract] AND "salvador"[Title/Abstract] | 13 |
|  | "gene"[Title/Abstract] AND "interaction"[Title/Abstract] AND "Honduras"[Title/Abstract] | 4 |
|  | "gene"[Title/Abstract] AND "interaction"[Title/Abstract] AND "Guatemala "[Title/Abstract] | 6 |
|  | ((gene) AND (interaction)) AND (latin) | 210 |
|  | ((((polymorphism OR gene OR SNP OR single nucleotide polymorphism OR genetic variation OR genetic variant) AND ("gene-diet interaction" OR "diet-gene interaction" OR SNP-diet interaction OR diet-SNP interaction OR "gene-nutrient interaction" OR "nutrient-gene interaction" OR "gene-lifestyle interaction" OR "gene-environment interaction")) AND (carbohydrate OR protein OR fat OR fibre OR sugar OR SFA OR saturated fat OR monounsaturated fat OR polyunsaturated fat OR MUFA OR PUFA OR Mediterranean diet OR Nordic diet OR B12 OR vitamin D OR amino acids OR polyphenols OR egg intake OR caffeine intake OR green tea OR alcohol intake OR meat intake OR energy intake OR physical activity level OR social factors OR socioeconomic)) AND (Obesity OR weight OR BMI OR waist circumference OR waist hip ratio OR hip circumference OR adiposity OR diabetes OR fasting glucose OR insulin OR HbA1c OR cardiovascular disease OR coronary heart disease OR ischaemic heart disease OR stroke OR lipids OR HDL OR LDL OR VLDL OR total cholesterol OR triglycerides OR triacylglycerol OR blood lipids OR serum lipids OR metabolic syndrome)) AND (Latin American OR Caribbean OR Haiti OR Cuba OR Dominican Republic OR Jamaica OR Trinidad and Tobago OR Bahamas OR Barbados OR Saint Lucia OR Grenada OR St. Vincent and Grenadines OR Antigua and Barbuda OR Dominica OR Saint Kitts & Nevis OR Mexico OR Guatemala OR Honduras OR Nicaragua OR El Salvador OR Costa Rica OR Panama OR Belize OR Brazil OR Colombia OR Argentina OR Peru OR Venezuela OR Chile OR Ecuador OR Bolivia OR Paraguay OR Uruguay OR Guyana OR Suriname OR Guadeloupe OR Martinique OR French Guiana OR Curacao OR Aruba OR Sint Maarten OR Caribbean Netherlands OR Cayman Islands OR Turks and Caicos OR British Virgin Islands OR Anguilla OR Montserrat OR Falkland Islands OR Puerto Rico OR U.S. Virgin Islands)" | 1,948 |
| Science Direct | Title, abstract, keywords: genetic AND interaction AND diet AND latin | 6 |
|  | Title, abstract, keywords: gene-diet interaction AND latin | 5 |
|  | Title, abstract, keywords: gene AND interaction AND latin | 29 |
|  | Title, abstract, keywords: gene AND interaction AND caribbean | 22 |
| SciELO | (ab:(*genetic)) AND (ab:(interaction)) AND (ab:(latin)) | 7 |
|  | (ab:(gen )) AND (ab:(interaccion)) | 74 |
|  | (ab:(*genetic)) AND (ab:(interaction)) AND (ab:(caribbean)) | 3 |
| Scopus | ( ALL ( gene ) AND TITLE-ABS-KEY ( interaction* ) AND TITLE-ABS-KEY ( latin* ) ) | 771 |
|  | ( ALL ( gene ) AND TITLE-ABS-KEY ( interaction* ) AND TITLE-ABS-KEY ( caribbean* ) ) | 414 |
|  | ( ALL ( gene ) AND TITLE-ABS-KEY ( interaction* ) AND TITLE-ABS-KEY ( diet* ) AND TITLE-ABS-KEY ( latin ) ) | 133 |
|  | ( ALL ( gene ) AND TITLE-ABS-KEY ( interaction* ) AND TITLE-ABS-KEY ( haiti ) ) | 9 |
|  | ( ALL ( gene ) AND TITLE-ABS-KEY ( interaction* ) AND TITLE-ABS-KEY ( cuba ) ) | 89 |
|  | ( ALL ( gene ) AND TITLE-ABS-KEY ( interaction ) AND TITLE-ABS-KEY ( Dominican Republic ) ) | 32 |
|  | ( ALL ( gene ) AND TITLE-ABS-KEY ( interaction* ) AND TITLE-ABS-KEY ( jamaica ) ) | 46 |
|  | ( ALL ( gene ) AND TITLE-ABS-KEY ( interaction ) AND TITLE-ABS-KEY (Trinidad and Tobago) ) | 27 |
|  | ( ALL ( gene ) AND TITLE-ABS-KEY ( interaction ) AND TITLE-ABS-KEY (Bahamas) ) | 27 |
|  | ( ALL ( gene ) AND TITLE-ABS-KEY ( interaction ) AND TITLE-ABS-KEY (Barbados) ) | 19 |
|  | (ALL ( gene ) AND TITLE-ABS-KEY ( interaction ) AND TITLE-ABS-KEY (Saint Lucia) ) | 2 |
|  | ( ALL ( gene ) AND TITLE-ABS-KEY ( interaction ) AND TITLE-ABS-KEY (Grenada) ) | 8 |
|  | ( ALL ( gene ) AND TITLE-ABS-KEY ( interaction ) AND TITLE-ABS-KEY (St. Vincent & Grenadines) ) | 0 |
|  | ( ALL ( gene ) AND TITLE-ABS-KEY ( interaction ) AND TITLE-ABS-KEY (Antigua and Barbuda) ) | 0 |
|  | ( ALL ( gene ) AND TITLE-ABS-KEY ( interaction ) AND TITLE-ABS-KEY (Dominica) ) | 24 |
|  | ( ALL ( gene ) AND TITLE-ABS-KEY ( interaction ) AND TITLE-ABS-KEY (Saint Kitts & Nevis) ) | 0 |
|  | ( ALL ( gene ) AND TITLE-ABS-KEY ( interaction ) AND TITLE-ABS-KEY (Mexico) ) | 1242 |
|  | ( ALL ( gene ) AND TITLE-ABS-KEY ( interaction ) AND TITLE-ABS-KEY (Guatemala) ) | 55 |
|  | ( ALL ( gene ) AND TITLE-ABS-KEY ( interaction ) AND TITLE-ABS-KEY (Honduras) ) | 26 |
|  | ( ALL ( gene ) AND TITLE-ABS-KEY ( interaction ) AND TITLE-ABS-KEY (Nicaragua) ) | 25 |
|  | ( ALL ( gene ) AND TITLE-ABS-KEY ( interaction ) AND TITLE-ABS-KEY (El Salvador) ) | 110 |
|  | ( ALL ( gene ) AND TITLE-ABS-KEY ( interaction ) AND TITLE-ABS-KEY (Costa Rica) ) | 154 |
|  | ( ALL ( gene ) AND TITLE-ABS-KEY ( interaction ) AND TITLE-ABS-KEY (Panama) ) | 160 |
|  | ( ALL ( gene ) AND TITLE-ABS-KEY ( interaction ) AND TITLE-ABS-KEY (Belize) ) | 32 |
|  | ( ALL ( gene ) AND TITLE-ABS-KEY ( interaction ) AND TITLE-ABS-KEY (Brazil) ) | 2238 |
|  | ( ALL ( gene ) AND TITLE-ABS-KEY ( interaction ) AND TITLE-ABS-KEY (Colombia) ) | 297 |
|  | ( TITLE-ABS-KEY ( gene ) AND TITLE-ABS-KEY ( interaction* ) AND TITLE-ABS-KEY ( argentina ) ) | 204 |
|  | (TITLE-ABS-KEY ( gene ) AND TITLE-ABS-KEY ( interaction ) AND TITLE-ABS-KEY (Peru) ) | 88 |
|  | (TITLE-ABS-KEY ( gene ) AND TITLE-ABS-KEY ( interaction ) AND TITLE-ABS-KEY (Venezuela) ) | 61 |
|  | ( TITLE-ABS-KEY ( gene ) AND TITLE-ABS-KEY ( interaction ) AND TITLE-ABS-KEY (Chile) ) | 177 |
|  | ( TITLE-ABS-KEY ( gene ) AND TITLE-ABS-KEY ( interaction ) AND TITLE-ABS-KEY (Ecuador) ) | 63 |
|  | ( TITLE-ABS-KEY ( gene ) AND TITLE-ABS-KEY ( interaction ) AND TITLE-ABS-KEY (Bolivia) ) | 26 |
|  | ( TITLE-ABS-KEY ( gene ) AND TITLE-ABS-KEY ( interaction ) AND TITLE-ABS-KEY (Paraguay) ) | 17 |
|  | ( TITLE-ABS-KEY ( gene ) AND TITLE-ABS-KEY ( interaction ) AND TITLE-ABS-KEY (Uruguay) ) | 35 |
|  | ( TITLE-ABS-KEY ( gene ) AND TITLE-ABS-KEY ( interaction ) AND TITLE-ABS-KEY (Guyana) ) | 9 |
|  | ( TITLE-ABS-KEY ( gene ) AND TITLE-ABS-KEY ( interaction ) AND TITLE-ABS-KEY (Suriname) ) | 5 |
| Taylor & Francis Online | [Abstract: gene] AND [All: interaction] AND [All: latin] | 733 |
| MEDLINE (EBSCOhost) | [AB gene AND AB interact AND AB latin](https://web.p.ebscohost.com/ehost/breadbox/search?term=AB%20gene%20AND%20AB%20interact%20AND%20AB%20caribbean&sid=e2a596df-4b94-4188-ad58-6c59e953f92a%40redis&vid=70) | 0 |
|  | [AB gene AND AB interact AND AB caribbean](https://web.p.ebscohost.com/ehost/breadbox/search?term=AB%20gene%20AND%20AB%20interact%20AND%20AB%20caribbean&sid=e2a596df-4b94-4188-ad58-6c59e953f92a%40redis&vid=70) | 0 |
| Cochrane trails | gene in Title Abstract Keyword AND "interaction" in Title Abstract Keyword AND "Latin" in Title Abstract Keyword - (Word variations have been searched) | 7 |
|  | gene in Title Abstract Keyword AND "interaction" in Title Abstract Keyword AND "caribbean" in Title Abstract Keyword - (Word variations have been searched) | 1 |
| ERIC (EBSCOhost) | [AB gene AND AB interact AND AB latin](https://web.p.ebscohost.com/ehost/breadbox/search?term=AB%20gene%20AND%20AB%20interact%20AND%20AB%20caribbean&sid=e2a596df-4b94-4188-ad58-6c59e953f92a%40redis&vid=70) | 0 |
| LILACS | gen [Palavras do resumo] and interacción [Palavras do resumo] | 77 |
|  | interação [Palabras del resumen] and obesidade [Palabras del resumen] | 114 |
|  | interação [Palabras del resumen] and genética [Palabras del resumen] and diabetes [Palabras del resumen] | 23 |
|  | interação [Palabras del resumen] and genética [Palabras del resumen] and cardiovascular [Palabras del resumen] | 33 |
| IBECS | gen AND interacción | 80 |
| Google Scholar | genetic* interaction latin* "gene-interaction" | 4,472 |

# Section 2 – Risk of bias assessment

Appraisal tool for Cross-sectional studies (AXIS)

Introduction

1. Were the aims/ Objectives of the study clear?

Methods

1. Was the study design appropriate for the stated aim(s)?
2. Was the sample size justified?
3. Was the target/reference population clearly defined? (Is it clear who the research was about?
4. Was the sample frame taken from an appropriate population base so that it closely represented the target/reference population under investigation?
5. Was the selection process likely to select subjects/participants that were representative of the target/reference population under investigation?
6. Were measures undertaken to address and categorize non-responders?
7. Were the risk factor and outcome variables measured appropriate to the aims of the study?
8. Were the risk factor and outcome variables measured correctly using instruments/ measurements that had been trialled, piloted or published previously? (Only dietary, nutritional, physical activity assessment were evaluated)
9. Is it clear what was used to determined statistical significance and/or precision estimates? (e.g., p-values, CIs)
10. Were the methods (including statistical methods) sufficiently described to enable them to be repeated?

Results

1. Were the basic data adequately described?
2. Does the response rate raise concerns about non-response bias?
3. If appropriate, was information about non-responders described?
4. Were the results internally consistent?
5. Were the results for the analyses described in the methods, presented?

Discussion

1. Was the author´s discussion and conclusions justified by the results?
2. Were the limitations of the study discussed?
3. Were there any funding sources or conflicts that may affect the authors interpretations of the results?
4. Was ethical approval or consent of participants attained?

**Table S2. Summary Outcome of Assessment with the Appraisal Tool for Cross-Sectional Studies (AXIS)**

|  | Vilella et al 2017 | Young et al 2016 | Valadez et al, 2020 | Campos et al, 2000 | Andrade et al 2010 | Corella et al, 2009 | Brown et al 2003 | Ma et al, 2014 | Lai et al 2010 | Zheng et al 2014 | Prieto et al 2019 | Davis et al, 2010 | Dumitrescu et al 2012 | Oliveira et al 2017 | Ramos-Lopez et al 2019 | Paula et al 2010, | Schreiber et al 2013 | Alsulami et al 2021 | Oki et al 2017 | Norde et al 2016, | Torres-Sánchez et al 2006 | Hidalgo et al 2011 | Fujii et al 2019 | Tellechea et al 2009 | Barcelos et al 2015 | Campos-Perez et al 2020 | Carvalho et al 2019 | Fiegenbaum et al 2003 | Fiegenbaum et al 2007 | Garcia-Garcia et al 2014 | Giovanella et al 2020 | Norde et al 2018 | Ochoa-Martinez et al 2017 | Ochoa-Martinez et al 2021 | Oki et al 2016 |
| --- | --- | --- | --- | --- | --- | --- | --- | --- | --- | --- | --- | --- | --- | --- | --- | --- | --- | --- | --- | --- | --- | --- | --- | --- | --- | --- | --- | --- | --- | --- | --- | --- | --- | --- | --- |
|  | Y | Y | Y | Y | Y | Y | Y | Y | Y | Y | Y | Y | Y | Y | Y | Y | Y | Y | Y | Y | Y | Y | Y | Y | Y | Y | Y | Y | Y | Y | Y | Y | Y | Y | Y |
|  | Y | N | Y | Y | Y | Y | Y | Y | Y | Y | Y | Y | Y | Y | Y | Y | Y | Y | Y | Y | Y | Y | Y | Y | Y | Y | Y | Y | Y | Y | Y | Y | Y | Y | Y |
|  | Y | Y | Y | Y | Y | Y | Y | N | Y | Y | Y | Y | Y | Y | Y | Y | Y | Y | Y | Y | Y | Y | Y | Y | Y | Y | Y | Y | Y | Y | Y | Y | Y | Y | Y |
|  | Y | N | Y | Y | Y | Y | Y | Y | Y | Y | Y | N | Y | Y | Y | Y | Y | Y | Y | Y | Y | Y | Y | Y | Y | Y | Y | Y | Y | Y | Y | Y | Y | Y | Y |
|  | Y | N | Y | Y | Y | Y | Y | N | Y | Y | Y | N | Y | Y | Y | Y | Y | Y | Y | Y | Y | Y | Y | Y | Y | Y | Y | Y | Y | Y | Y | Y | Y | Y | Y |
|  | Y | Y | Y | Y | N | Y | Y | Y | Y | Y | Y | N | Y | Y | Y | Y | Y | Y | Y | Y | Y | Y | Y | Y | Y | - | Y | N | Y | Y | Y | Y | Y | Y | Y |
|  | Y | Y | Y | Y | Y | Y | Y | Y | Y | Y | Y | Y | Y | Y | Y | Y | Y | Y | Y | Y | Y | Y | Y | Y | Y | Y | Y | Y | Y | Y | Y | Y | Y | Y | Y |
|  | Y | N | N | Y | Y | Y | Y | Y | Y | Y | Y | Y | Y | Y | Y | Y | Y | Y | Y | Y | Y | Y | Y | Y | Y | Y | Y | Y | Y | Y | Y | Y | Y | Y | Y |
|  | Y | N | Y | Y | Y | Y | Y | N | Y | Y | Y | Y | Y | Y | Y | Y | Y | Y | Y | Y | Y | - | Y | - | Y | Y | Y | Y | Y | Y | Y | Y | Y | Y | Y |
|  | Y | Y | Y | Y | N | Y | Y | Y | Y | Y | Y | Y | Y | Y | Y | Y | Y | Y | Y | Y | Y | Y | Y | Y | Y | Y | Y | Y | - | Y | Y | Y | Y | Y | Y |
|  | Y | Y | Y | Y | Y | Y | Y | Y | Y | Y | Y | Y | Y | Y | Y | Y | Y | Y | Y | Y | Y | Y | Y | Y | Y | Y | Y | Y | Y | Y | Y | Y | Y | Y | Y |
|  | Y | Y | Y | Y | Y | Y | Y | Y | Y | Y | Y | Y | Y | Y | Y | Y | Y | Y | Y | Y | Y | Y | Y | Y | Y | Y | Y | Y | Y | Y | Y | Y | Y | Y | Y |
|  | Y | N | N | N | N | N | N | N | N | N | N | N | N | N | N | N | N | N | N | N | N | N | N | N | N | N | N | N | N | N | N | N | N | N | N |
|  | N | Y | Y | Y | Y | Y | Y | y | Y | Y | Y | Y | Y | Y | Y | Y | Y | Y | Y | Y | Y | Y | Y | Y | Y | Y | Y | Y | Y | Y | Y | Y | Y | Y | Y |
|  | Y | Y | Y | Y | - | Y | Y | y | Y | - | Y | Y | Y | Y | Y | Y | Y | Y | Y | Y | Y | Y | Y | Y | Y | Y | Y | Y | Y | Y | Y | Y | Y | Y | Y |
|  | Y | Y | Y | Y | Y | Y | Y | y | Y | Y | Y | Y | Y | Y | Y | Y | Y | Y | Y | Y | Y | Y | Y | Y | Y | Y | Y | Y | Y | Y | Y | Y | Y | Y | Y |
|  | Y | Y | Y | Y | Y | Y | Y | Y | Y | Y | Y | Y | Y | Y | Y | Y | Y | Y | Y | Y | Y | Y | Y | Y | Y | Y | Y | Y | Y | Y | Y | Y | Y | Y | Y |
|  | Y | Y | Y | Y | Y | Y | Y | Y | Y | Y | Y | Y | Y | Y | Y | Y | Y | Y | Y | Y | Y | Y | Y | Y | N | Y | Y | Y | Y | Y | Y | Y | Y | Y | Y |
|  | N | N | N | N | N | N | N | N | N | N | N | N | N | N | N | N | N | N | N | N | N | N | N | N | N | N | N | N | N | N | N | N | N | N | N |
|  | Y | Y | Y | Y | Y | Y | Y | Y | Y | Y | Y | Y | Y | Y | Y | Y | Y | Y | Y | Y | Y | Y | Y | Y | Y | Y | Y | Y | Y | Y | Y | Y | Y | Y | Y |

**Note: Numbered questions are listed apart.**

**Continuation of Table S2.**

|  | Study 1 | Orozco et al 2014 | Muñoz et al 2017 | Yu et al 2017 | Alathari et al 2022 | Dominguez-Rey et al 2015 | Costa-Urrutia et al 2018 | Todendi et al 2021 | Freire et al 2017 | Sehn et al 2022 | Mattei et al 2011 | Mattei et al 2009 | Surendran et al 2019 | Ma et al 2012 |
| --- | --- | --- | --- | --- | --- | --- | --- | --- | --- | --- | --- | --- | --- | --- |
| Introduction | | | | | | | | | | | | |  |  |
|  | Y | Y | Y | Y | Y | Y | Y | Y | Y | Y | Y | Y | Y | Y |
| Methods | | | | | | | | | | | | |  |  |
|  | Y | Y | Y | Y | Y | Y | Y | Y | Y | Y | Y | Y | Y | Y |
|  | Y | Y | Y | Y | Y | Y | Y | Y | Y | Y | Y | Y | Y | Y |
|  | Y | Y | Y | Y | Y | Y | Y | Y | Y | Y | Y | Y | Y | Y |
|  | Y | Y | Y | Y | Y | Y | Y | Y | Y | Y | Y | Y | Y | Y |
|  | Y | Y | Y | Y | Y | Y | Y | Y | Y | Y | Y | Y | Y | Y |
|  | Y | Y | Y | Y | Y | Y | Y | Y | Y | Y | Y | Y | Y | Y |
|  | Y | Y | Y | Y | Y | Y | Y | Y | Y | Y | Y | Y | Y | Y |
|  | Y | Y | Y | Y | Y | Y | Y | Y | Y | Y | Y | Y | Y | Y |
|  | Y | Y | Y | Y | Y | Y | Y | Y | Y | Y | Y | Y | Y | Y |
|  | Y | Y | Y | Y | Y | Y | Y | Y | Y | Y | Y | Y | Y | Y |
| Results | | | | | | | | | | | | |  |  |
|  | N | N | N | N | N | N | N | N | N | N | N | N | N | N |
|  | - | - | - | - | - | - | - | - | - | - | - | - | - | - |
|  | Y | Y | Y | Y | Y | Y | Y | Y | Y | Y | Y | Y | Y | Y |
|  | Y | Y | Y | Y | Y | Y | Y | Y | Y | Y | Y | Y | Y | Y |
|  | N | N | N | N | N | N | N | N | N | N | N | N | N | N |
| Discussion | | | | | | | | | | | | |  |  |
|  | Y | Y | Y | Y | Y | Y | Y | Y | Y | Y | Y | Y | Y | Y |
|  | Y | Y | Y | Y | Y | Y | Y | Y | Y | Y | Y | Y | Y | Y |
|  | N | N | N | N | N | N | N | N | N | N | N | N | N | N |
|  | Y | Y | Y | Y | Y | Y | Y | Y | Y | Y | Y | Y | Y | Y |

**Note: Numbered questions are listed apart.**

**Table S3. Assessment with the Comments Appraisal Tool for Cross-Sectional Studies**

|  | **Introduction** | **Method** | | | | | | | | | | **Results** | | | | | **Discussion** | | | |
| --- | --- | --- | --- | --- | --- | --- | --- | --- | --- | --- | --- | --- | --- | --- | --- | --- | --- | --- | --- | --- |
|  | 1. | 2. | 3 | 4. | 5. | 6. | 7. | 8. | 9. | 10. | 11 | 12. | 13 | 14. | 15. | 16. | 17. | 18. | 19. | 20. |
| Vilella et al 2017 | Y | Y | Y | Y | Y | Y | Y | N | Y | Y | Y | Y | Y | Y | Y | Y | Y | Y | N | Y |
| **8**. 24-h diet recall and global BMI values to establish percentiles in which participants were fixed into are both measures leading to bias  **13**. > 5 % of the data were measing | | | | | | | | | | | | | | | | | | | | |
| Young et al 2016 | Y | N | Y | N | N | Y | Y | N | Y | Y | Y | Y | N | Y | Y | Y | Y | Y | N | Y |
| **2**. No methodology available in full text  **4.** Only characteristics "Latino children and adults"  **5**. N/A  **8**. Physical activity was measured via a dichotomic question | | | | | | | | | | | | | | | | | | | | |
| Andrade et al 2010 | Y | Y | Y | Y | Y | **N** | Y | Y | Y | **N** | Y | Y | N | Y | Y | Y | Y | Y | N | Y |
| **6.** In Brazil has the highest admixture index in the world an only European ancestry population were included.  **10**. Not clarified in the methods | | | | | | | | | | | | | | | | | | | | |
| Ma et al, 2014 | Y | Y | N | Y | N | Y | Y | Y | N | Y | Y | Y | N | Y | Y | Y | Y | Y | N | Y |
| **5.** Subgroup representing a minority  **9.** Food frequency questionnaire not adapted to the subgroup´s gastronomic culture | | | | | | | | | | | | | | | | | | | | |
| Zheng et al 2014 | Y | Y | Y | Y | Y | Y | Y | Y | Y | Y | Y | Y | N | Y | - | Y | Y | Y | N | Y |
| **15**. Data available in graphs not in tables hence consistency of results was no possible to determine | | | | | | | | | | | | | | | | | | | | |
| Davis et al, 2010 | Y | Y | Y | N | N | N | Y | Y | Y | Y | Y | Y | N | Y | Y | Y | Y | Y | N | Y |
| **4.** Samples from other studies, brief description, "Hispanic children and adolescents"  **5**. The sample was taken from protocols and measures conducted by a third party during the past 6 years.  **6.** Hispanic children recruited from schools, community centers, health clinics, health fairs via word of mouth, flyers and in-person contact. | | | | | | | | | | | | | | | | | | | | |
| Barcelos et al 2015 | Y | Y | Y | Y | Y | Y | Y | Y | Y | Y | Y | Y | N | Y | Y | Y | Y | N | N | Y |
| **18.** No limitations were discussed | | | | | | | | | | | | | | | | | | | | |

**Note: Questions are listed above, Table S2 summarises Table S3.**

**Table S4. Assessment using the Risk of Bias in Non-Randomized Studies – of Interventions (ROBINS-I)**

| ROBINS-I assessment | **1.1 Is there potential for confounding of the effect of exposure in this study? If N or PN to 1.1: the study can be considered to be at low risk of bias due to confounding and no further signalling questions need be considered Y / PY / PN / N** | | | | | | | | | |  |
| --- | --- | --- | --- | --- | --- | --- | --- | --- | --- | --- | --- |
| Smith et al 2008 | PN | | | | | | | | | |  |
| [Description] | "Dietary intake was assessed using a FFQ that was designed for and tested in this population". "The interactions were tested with control for potential confounders including age, sex, alcohol (never, past, current), smoking (never, past, current), physical activity, diabetes medications, and dietary fibre". | | | | | | | | | |  |
| Smith et al 2013 | PN | | | | | | | | | |  |
| [Description] | "The modified FFQ, which includes foods commonly consumed by Hispanics and open-ended portion sizes, more accurately estimated nutrients and energy intake in older Hispanics than the original FFQ based on its improved correlation with dietary recall data" "The interactions were tested with control for potential confounders including age, sex, alcohol intake (g/d), smoking (current vs. never and former), physical activity, antiglycemic medication, and ancestral admixture." | | | | | | | | | |  |
| Moon et al 2017 | PN | | | | | | | | | |  |
| [Description] | "Conducted various sensitivity analyses and subgroup analyses. Used a DXA and measured weight to the nearest 0.1kg. | | | | | | | | | |  |
| Portillo et al 2022 | PN | | | | | | | | | |  |
| [Description] | Dietary questionnaire "applied by previously trained personnel" "Adjusted for possible confounders: adjusted for: age, sex, BMI, waist circumference, physical activity, schooling, socioeconomic level, smoking, consumptions: fruit, vegetables, sugar, processed meats, alcoholic beverages and % Amerindian ancestry" | | | | | | | | | |  |
| Torres Sánchez et al 2014 | PN | | | | | | | | | |  |
| [Description] | Uses a validated questionnaire carried out by trained personnel, and the methodology is being replicated from 2 other studies. "The following known risk factors for foetal development were selected as potential confounders: maternal age (years), height (cm), education (years), paid occupation (yes/no), parity (none/1–2) and body mass index during the first trimester of pregnancy (kg/m2) and usage of vitamin supplements during pregnancy." "After adjusting separately for potential confounding variables in each model and correcting by bootstrap resampling..." | | | | | | | | | |  |
| Horta et al 2018 | PN | | | | | | | | | |  |
| [Description] | Used DXA and stadiometer with accuracy, visceral and subcutaneous abdominal fat thickness were estimates with ultrasound. WC was measured twice. It acknowledges possible confounders and adjusts. | | | | | | | | | |  |
| Guevara-Cruz et al 2014 | PN | | | | | | | | | |  |
| [Description] | Methodology explained thoroughly . Nutritionist assigned for the follow up. Analysis stratified and visible in tables. | | | | | | | | | |  |
| Lopez-Ortiz et al 2016 | PN | | | | | | | | | |  |
| [Description] | "Dietary evaluations were carried out using 24-h recall questionnaires and 3-d dietary records, and these were applied to evaluate 2 weekdays and 1 weekend day using standardised measures of food portions; the information was collected by direct interview." "We compared changes in end points across genotype groups according to diet groups at 8 weeks. To assess the effects of genotype, dietary treatment and their interaction, we used a general linear model (GLM) repeated-measures analysis, and age was included in the model as a covariate." | | | | | | | | | |  |
| Sir-Petermann et al 2004 | PN | | | | | | | | | |  |
| [Description] | Pre-established protocol, and clear inclusion and exclusion criteria, | | | | | | | | | |  |
| Prieto et al 2016 | PN | | | | | | | | | |  |
| [Description] | Use of previously used and validated scale “lifestyle cardiovascular risk score (LCRS)”. Clear adjustment in analysis “We examined interaction and joint associations for each component of the LCRS separately and controlling for each other (i.e., adjusted for the other lifestyles in the LCRS). | | | | | | | | | |  |
| Yang et al 2007 | PN | | | | | | | | | |  |
| [Description] | “Trained personnel visited all study participants”. “Generalized linear models adjusted for age, sex, body mass index, and physical activity were used to report the relationship between plasma lipid levels, saturated fat intake, and APOE genotype”. | | | | | | | | | |  |
| Ruiz-Narvaez et al 2007 | PN | | | | | | | | | |  |
| [Description] | “Trained personnel visited all study participants at their homes for data collection, biological specimen collection, and anthropometric measurements.” | | | | | | | | | |  |
| Hartiala et al 2012 | PN | | | | | | | | | |  |
| [Description] | Study that aims to replicate previous observations by another study. “Trained personnel visited all study participants at their homes for data collection”. Use of fully adjusted model including age, sex, county of residence, % of total energy from fat, smoking, household income, history of diabetes, hypertension, or hypercholesteremia, obesity, and family history of MI. | | | | | | | | | |  |
| Zheng et al 2016 | PN | | | | | | | | | |  |
| [Description] | Clear aim. Use of culturally adapted food-frequency questionnaire (FFQ) “FFQ that was developed and validated specifically for the Costa Rican population”. Clear assessment of covariates plus transparency in statistical analysis. | | | | | | | | | |  |
| Cornelis et al 2007 | PN | | | | | | | | | |  |
| [Description] | Clear objective, description of cases and matched controlled. Transparency in statistical analysis, and adjustment for confounding variables. Besides use of trained personnel and closed ended questionnaires: “ All data were collected by trained fieldworkers during an interview using 2 questionnaires consisting of closed-ended questions” | | | | | | | | | |  |
| Sen-Banerjee et al 2000 | PN | | | | | | | | | |  |
| [Description] | Clear purpose of the study, description of cases and controls, inclusion and exclusion criteria, transparency in statistical analysis, and adjustment for covariates. However, data sources from self-reported diabetes and hypertension, but it was validated using standardized definitions. | | | | | | | | | |  |
| Costa-Urrutia et al 2017 | PN | | | | | | | | | |  |
| [Description] | Clear aim, inclusion and exclusion criteria, transparent statistical analysis and adjusted for covariates. Furthermore, pre-established power calculations. | | | | | | | | | |  |
| Guevara-Cruz et al 2013 | PN | | | | | | | | | |  |
| [Description] | Clear aim, inclusion and exclusion criteria, transparent statistical analysis and adjustment for covariates, consideration of limitations in the study “the determination of only a single gene polymorphism and the subjects participating in this study belong to a specific ethnic group” | | | | | | | | | |  |
| Nascimento et al 2018 | PN | | | | | | | | | |  |
| [Description] | Appropriate methods for aim, clear inclusion and exclusion criteria, adjustment for covariates and transparent statistical test, consideration of limitations “This study was limited to analysis of the biochemical variable sin these individuals, some of the, were analysed in a previous study that was limited to anthropometric variable analysis.” | | | | | | | | | |  |
| Nascimento et al 2019 | PN | | | | | | | | | |  |
| [Description] | Clear study design. Nevertheless, the small sample size may have influenced the identification of minor effects. | | | | | | | | | |  |
| Cornelis et al 2004 | PN | | | | | | | | | |  |
| [Description] | Clear aim and study design. Inclusion of univariate and multivariate analysis with adjustment for confounding variables. | | | | | | | | | |  |
| El-Sohemy et al 2007 | PN | | | | | | | | | |  |
| [Description] | Case-control study clear inclusion and exclusion criteria, appropriate study design, adjustment for confounding variables, and use of validated and culturally adapted FFQ | | | | | | | | | |  |
|  | **1.1 Is there potential for confounding of the effect of exposure in this study? If N or PN to 1.1: the study can be considered to be at low risk of bias due to confounding and no further signalling questions need be considered Y / PY / PN / N** | If Y/PY to 1.1, answer 2.1 and 1.3 to determine whether there is a need to assess time-varying confounding: | **1.2. If Y or PY to 1.1: Was the analysis based on splitting, follow up time according to exposure received?** | If N or PN to 1.2, answer questions 1.4 to 1.6, which relate to baseline confounding | **1.3. If Y or PY to 1.2: Were exposure discontinuations or switches likely to be related to factors that are prognostic for the outcome?** | If N or PN to 1.3, answer questions 1.4 to 1.6, which relate to baseline confounding | Bias in selection of participants into the study | **2.1. Was selection of participants into the study (or into the analysis) based on variables measured after the start of the exposure?** | If N or PN to 2.1 go to 2.4 | **2.4 Do start of follow-up and start of exposure coincide for most participants?** | |
| Correa et al 2013 | PY |  | Y |  | Y |  |  | N |  | PY | |
| [Description] | No established calibration of instruments, however rigorous methods for estimating alcohol consumption. |  |  |  | "Completers, lost because of drop out, lost because of death" and it was measuring alcohol intake. Deaths and drop out could have been related. |  |  |  |  | "The baseline cohort population consisted of all residents aged ≥60 years on 1 January 1997, who were identified by means of a complete census of the town." | |
